# HeRZ: Hierarchical Recursive Zero-Knowledge Verification for Canonical and Auditable Asset-State Evolution in Blockchain-Cyber-Physical Systems

**DOI:** 10.3390/s26196226

**Published:** 2026-09-30

**Authors:** Ningyuan Chen, Yibei Lin, Siu-Yeung Cho, Yu Zheng

**Affiliations:** 1 College of Information and Intelligence Engineering, Zhejiang Wanli University, No. 8, Qianhu South Road, Yinzhou District, Ningbo 315100, China; 2023013900@zwu.edu.cn; 2 Department of Electrical and Electronic Engineering, The Hong Kong Polytechnic University, Hung Hom, Kowloon, Hong Kong, China; davidcho.ieee@gmail.com (S.-Y.C.); yuz339@connect.hku.hk (Y.Z.); 3 Oriensol Technology Company Limited, Hung Hom, Kowloon, Hong Kong, China

**Keywords:** blockchain-cyber-physical systems, blockchain-powered sensing, internet of things, sensor data integrity, state-evolution integrity, zero-knowledge proofs, historical auditability

## Abstract

Blockchain-Cyber-Physical Systems (BCPSs) use sensor-generated IoT events to update ledger representations of physical assets. Authenticating individual reports does not by itself establish an admissible trajectory from the canonical predecessor or authenticate a selected historical interval. We formulate Verifiable State-Evolution Integrity (VSEI): accepted states must be reachable through ordered, device-bound, policy-compliant trajectories. HeRZ realizes this requirement through a BCPS-specific relation combining event authentication, physical guards, deterministic updates, and temporal linkage. Recursive folding preserves the relation in a fixed-width incremental proof state, while a KZG/Groth16 Decider exposes the interval boundaries. A stateful EVM wrapper atomically consumes the canonical predecessor for forward settlement and verifies retained intervals between anchored checkpoints without publishing intermediate event records. The implementation supports both paths, producing 384-byte compressed proofs and 1156-byte settlement calldata. Mean complete online peak memory remains 27.92–28.68 GiB over 100–2000 events. At 2000 events, HeRZ reduces peak memory by 34.1% relative to the equivalent stateful FlatZK baseline, at 3.63 times its online time. Layered tests and anchored audits validate the implemented state semantics. One parameter set and deployed verifier accept different interval lengths, supporting incremental construction and selective historical auditing within a fixed protocol version. HeRZ thus provides an executable path from authenticated sensor events to canonically settled and retrospectively auditable asset histories.

## 1. Introduction

The evolution of blockchain technology has extended beyond native digital assets to the tokenization of tangible resources, a paradigm often referred to as Real-World Assets (RWA) or Decentralized Physical Infrastructure Networks (DePINs) [1,2]. By representing sensor-observed physical assets such as photovoltaic installations, supply-chain inventories, and environmental monitoring infrastructure on distributed ledgers, these systems aim to enhance liquidity, transparency, and automated governance. However, the integration of immutable ledgers with dynamic physical systems introduces a fundamental contradiction: blockchain states are discrete and final once committed, while physical states are continuous and evolve at high frequency.

Current blockchain-enabled sensing systems primarily rely on blockchain oracles to bridge this gap [3,4,5]. Such snapshot-based designs suffer from three limitations. First, they depend on external trust in data relayers or hardware sources and cannot cryptographically verify the integrity of the state-evolution process itself. An attacker may therefore tamper with a bound event, reorder valid events, replay a settled trajectory, or submit a competing successor state without violating the semantics of an isolated report. The same limitation obstructs retrospective auditing: a verifier cannot select two historical checkpoints and establish that every intervening update followed the authorized evolution policy without retrieving the raw event stream. Second, directly synchronizing granular industrial IoT events to a Layer-1 blockchain is economically infeasible because of computation and storage costs [6]. Third, exposing high-frequency operational data on public ledgers leaks commercially sensitive information [7].

To address these challenges, we introduce HeRZ, a Hierarchical Recursive Zero-knowledge protocol for verifiable physical-state evolution in BCPSs. HeRZ changes the verification object: instead of authenticating an isolated sensor report or independently valid batch, it proves that one asset state is reachable from another through a device-authenticated, policy-constrained, and temporally linked trajectory. A compact step circuit defines one admissible transition, folding-based IVC preserves the same relation over any retained interval containing at least two transitions, and a KZG/Groth16 Decider exposes the interval boundaries as a succinct statement. This statement has two complementary uses. For forward settlement, the initial boundary must equal the currently finalized state, after which the accepted endpoint becomes the unique canonical successor. For retrospective audit, two previously anchored checkpoints delimit a historical interval whose intervening evolution can be verified without publishing its raw event records. The pipeline separates incremental event processing from blockchain settlement, retaining constant O(1) verifier complexity with respect to the number of folded events [8].

We call this combined requirement Verifiable State-Evolution Integrity (VSEI): a proof establishes an ordered, device-bound, policy-compliant path between its boundary states, and settlement consumes the current canonical predecessor exactly once. Ordinary state validity can establish that an endpoint satisfies a predicate without establishing this authenticated path or its canonical predecessor. VSEI specifies the combined application guarantee; it does not require recursion in principle. HeRZ uses recursion to implement that guarantee incrementally and to reconstruct proofs for retained historical intervals.

We implemented the step relation, complete Nova/CycleFold Decider, canonical settlement, and read-only audit interface, together with an equivalent stateful non-recursive baseline. Sixty formal online runs across three methods and four interval lengths include parameter loading and fresh complete proofs. HeRZ maintains fixed-width recursive state and 1156-byte settlement calldata; one deployed verifier accepts different lengths and anchored historical queries. At 2000 events, HeRZ uses 34.1% less peak memory than the equivalent FlatZK baseline at 3.63 times its online time, while FlatZK remains faster and more memory-efficient at smaller tested scales. Trace-derived controls and layered tests separately validate application predicates and canonical/audit behavior.

In summary, this paper makes the following four main contributions:

- **Verifiable State-Evolution Integrity:** We formulate BCPS state continuity as reachability over sensor-driven asset states. Every accepted segment contains only device-bound events, protocol-fixed physical guards, deterministic updates, and temporally linked history commitments. We establish path soundness for retained intervals of at least two transitions and canonical finality for state-advancing settlements under explicit cryptographic and contract-execution assumptions.
- **Hierarchical Relation-Preserving Proofs:** HeRZ realizes one BCPS-specific state-evolution relation across three execution domains: a per-event edge circuit establishes an admissible transition, recursive folding preserves the reachability claim across a selected interval with bounded incremental prover memory, and a succinct Decider exposes only the interval boundaries and constant-size verifier inputs.
- **Canonical Settlement and Historical Interval Audit:** A stateful Solidity wrapper consumes the proof’s initial and final five-field states atomically, making an accepted reachable endpoint the unique canonical successor and rejecting replayed or competing histories. The same boundary-parametric proof statement also supports read-only verification between anchored historical checkpoints, enabling selective lifecycle audits without publishing the intervening events.
- **End-to-End Implementation and Evaluation:** An equivalent stateful FlatZK baseline isolates the proving-architecture trade-off. Complete Decider measurements, retained-interval audits, trace-derived controls, and layered adversarial tests quantify setup, proving, memory, storage, and verification costs while checking event binding, policy enforcement, replay/fork resistance, and historical boundary authentication.

Together, the step relation, recursive folding, and stateful settlement preserve one cross-layer invariant from authenticated events to a canonical endpoint; boundary-parametric verification extends the same invariant to selected historical intervals.

Section 2 reviews related work; Section 3, Section 4 and Section 5 present the models, protocol, and security analysis; and Section 6, Section 7 and Section 8 report the evaluation, discussion, and conclusions.

## 2. Related Works

### 2.1. Blockchain Oracles and IoT Data Integrity

Blockchain-enabled CPS applications use digital twins and AI for urban energy management [9,10] but generally ingest physical data through gateways or APIs without proving consistency of the generating process. Oracle systems authenticate external inputs through voting or reputation [11,12,13,14] or hardware attestation [15]. Voting systems establish agreement rather than cryptographic correctness and remain exposed to collusion and identity concentration [16]; TEEs strengthen source authentication but transfer trust to hardware and attestation services. Neither class proves that a ledger state follows an ordered, policy-compliant physical trajectory.

**Figure 1:**
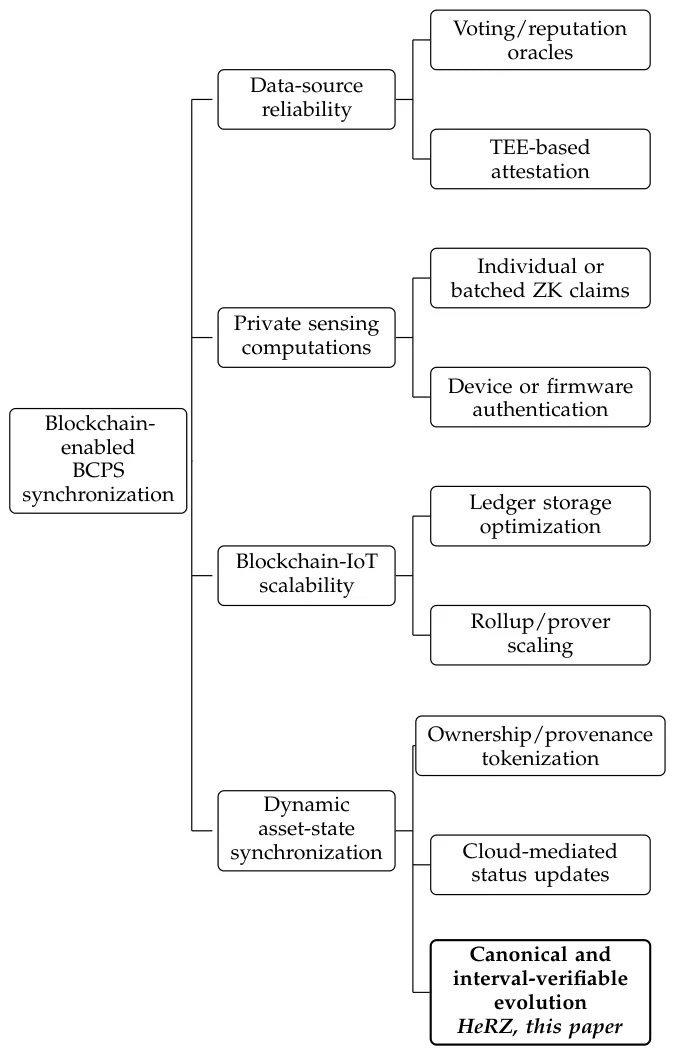
BCPS synchronization taxonomy and HeRZ’s position. References by category: voting/reputation oracles [3,4,5,13,14]; TEE-based attestation [15]; individual or batched ZK claims [17,18,19]; device or firmware authentication [17,18]; ledger storage optimization [6]; rollup/prover scaling [20,21,22,23]; ownership/provenance tokenization [24,25]; cloud-mediated status updates [26,27].

To position HeRZ, Figure 1 classifies prior BCPS blockchain studies by what they make verifiable: data sources, discrete privacy claims, infrastructure scalability, or dynamic asset-state synchronization. Within the dynamic asset-state branch, prior work mainly records ownership/provenance or cloud-mediated status updates, whereas HeRZ makes the evolution path itself verifiable for both canonical settlement and selected historical intervals.

Recent oracles improve source selection through zero-trust reputation, DAG consensus, or reinforcement learning [3,4,5], while blockchain-IoT ZK systems authenticate devices, aggregate signed sensing data, or verify privacy-preserving sensing computations [17,18,19]. These are distinct verification targets: authenticating a batch or sensing computation does not by itself specify a BCPS asset-state trajectory and its canonical predecessor. Granular on-chain records remain storage-intensive [6]. HeRZ instead compresses authenticated transitions between selected checkpoints into one statement for canonical advancement or retrospective audit.

### 2.2. Zero-Knowledge Architectures for Distributed and Constrained Systems

Nova makes IVC practical through folding [21], but leaves the application relation, witness trust boundary, and settlement semantics to the system designer. Pianist parallelizes proofs across clusters [22], while Ceno segments general-purpose zkVM execution [23]; both target broader proving workloads rather than a fixed, low-bandwidth BCPS transition relation.

Related IoT ZK systems verify firmware execution or privacy-preserving vehicular claims [17,28]. Burgos and Pustišek aggregate signed IoT data with Nova at edge servers and anchor batch metadata to a Layer-2 ledger [18], whereas Jia et al. use zero-knowledge arguments and Merkle commitments to make crowd-sensing aggregation verifiable [19]. The close connection to recursive IoT authentication is important: HeRZ’s distinction is not the use of Nova at an edge server but the definition and enforcement of the device-bound asset transition, canonical cross-batch consumption, and same-version interval boundaries as one application statement. HeRZ folds this relation and requires each advancing proof to consume the finalized state; the same boundary semantics support anchored interval audits.

### 2.3. Digital Twins and Dynamic Asset Management

TokenGreen and supply-chain frameworks record ownership or provenance as NFT metadata [24,25]; ERC-4907 separates user rights from ownership [29]; and dynamic-twin systems update asset metadata through cloud simulations or alerts [26,27]. These designs improve asset representation but retain an externally trusted update path. Consequently, the Garbage-In, Garbage-Out problem persists: an immutable ledger can preserve a spoofed sensor report or compromised cloud output without proving the physical-to-digital transition [30].

HeRZ addresses this persistent GIGO challenge by embedding the state transition logic directly into the cryptographic proof. Instead of relying on a black-box cloud simulation, HeRZ enables the asset-side prover to generate a succinct certificate that its ledger state is consistent with an authenticated, policy-constrained event trajectory. This does not establish absolute physical truth, but it closes the verification gap between authenticated observations and dynamic asset-state updates.

Figure 1 and Table 1 summarize the literature by verification target. Table 2 then compares the relevant protocol obligations explicitly. Existing work targets data-source reliability, discrete privacy claims, blockchain-IoT scalability, or externally updated asset representations. HeRZ instead proves the evolution path itself for canonical settlement or selective audit with constant verification overhead.

## 3. System Architecture

This section formalizes the system entities, trust assumptions, and threat model of the proposed protocol. It also gives a high-level overview of the HeRZ architecture and its secure synchronization of continuous physical events.

### 3.1. System Entities and Trust Assumptions

The HeRZ ecosystem comprises four entities with explicit computational and trust boundaries:

- **Sensors and Secure Elements:** Physical sensors produce measurements, while a TEE/SE protects the factory-provisioned secret $sk_{\mathrm{dev}}$ and authenticates each event. The hardware boundary is the root of trust for data origination.
- **Secure Per-Asset Edge Prover:** A gateway colocated with the asset executes the step relation and Nova folding inside the same protected trust boundary as the device secret. Because $sk_{\mathrm{dev}}$ is a private circuit witness, authenticated step evaluation and folding remain inside this protected gateway, while fog and cloud services operate on released state and proof material.
- **Untrusted Relay/Fog Service:** A fog or cloud service may buffer, schedule, and forward folded instances or final proofs, but it is not entrusted with $sk_{\mathrm{dev}}$ and does not execute the witness-dependent folding step. It may drop, delay, replay, or reorder messages; the on-chain canonical-state checks reject stale or competing settlements, although denial of service remains possible.
- **On-chain Verifier (Smart Contracts):** Deployed on a public blockchain, this entity verifies finalized proofs and maintains the canonical state. ***Trust Assumption:*** Consensus-finalized contract execution is trusted to apply the verifier and state-transition logic correctly; availability of off-chain events and proofs is not guaranteed by the contract.

The sensor and protected edge prover form one trusted acquisition domain sharing the device secret. A symmetric tag binds each acquired event to its device and order, and the blockchain verifies a proof that this same event satisfies the state-evolution relation. Because the prover is an authorized key holder, source authenticity relies on the integrity of this acquisition domain; tag verification is not independent attestation of a separate sensor against that prover.

**Definition** **1** (Device Binding, Policy Compliance, and Physical Truth)**.** *Device binding means that the event is authenticated within the registered acquisition domain. Policy compliance means that its fields satisfy the compiled predicates. Physical truth means correspondence to the actual environment, which additionally depends on sensing, calibration, and the acquisition domain’s integrity. HeRZ proves the first two properties along a linked trajectory. A correctly authenticated but fabricated value inside the policy envelope remains admissible to the relation; this is distinct from accepting an event that violates the relation.*

### 3.2. Threat Model

The threat model is defined by establishing the attack vectors the HeRZ protocol is designed to neutralize (in-scope) and the assumptions falling outside its protective boundaries (out-of-scope).

#### 3.2.1. The In-Scope Threats

- **Data Injection Attacks:** An event may be correctly authenticated yet violate the configured operating policy. HeRZ rejects such transitions by enforcing physical and logical predicates (Φ) inside the step circuit. The evaluated PV predicates define an admissible operating envelope, rather than a complete generative model of PV physics.
- **State Forking, Replay, and Historical Substitution:** An adversary attempts to replay older transitions, fork the asset’s history, or present an invalid segment as part of a historical audit. Within a proof interval, the folded relation requires step i+1 to consume the state produced by step *i* and binds both interval boundaries and the step count. Across state-advancing batches, the wrapper additionally requires the proof’s initial state to match the currently settled canonical state.
- **Data Decoupling and Equivocation:** An adversary attempts to pass the circuit validity checks using legitimate data but commits a malicious payload to the historical ledger. HeRZ neutralizes this by enforcing internal signal consistency. The arithmetic constraints dictate that the exact signal satisfying the physical predicates must be identical to the signal fed into the commitment function.
- **Privacy Leaks:** Competitors attempt to infer commercial secrets by analyzing on-chain verification data. HeRZ avoids directly publishing raw per-event values and auxiliary witnesses: the Groth16 Decider proves the folded relation without disclosing its private witness. This does not constitute a system-level transcript-privacy guarantee. The five-field boundary state $z_{N}$, including its cumulative value, event count, and latest logical slot, remains public, while the binding but non-hiding KZG commitments and openings disclose commitment data and challenge-point evaluations. These public values may permit aggregate or algebraic inference.

#### 3.2.2. The Out-of-Scope Threats

The protocol assumes that physical sensor destruction or hardware-level extraction of $sk_{\mathrm{dev}}$ is beyond the system’s scope. Defending against such hardware breaches requires specialized tamper-proof engineering, which remains orthogonal to the cryptographic protocol design.

### 3.3. Logical Time and Acquisition Completeness

The authenticated $\tau_{t}$ is a logical acquisition-slot index assigned by the trusted acquisition domain, not a blockchain timestamp or an independently attested wall clock. The circuit requires $q_{t+1}=q_{t}+1$ and $\tau_{t}={\bar{\tau }}_{t}+1$. Consequently, an omitted slot followed by its original later index fails, whereas buffering and later proving a complete sequence is allowed. A relay cannot reorder or renumber an existing authenticated sequence without invalidating its bindings. The protocol does not infer whether the acquisition domain failed to observe a physical event before assigning slots, nor does it measure clock drift.

The evaluated relation has no missing-reading marker or gap-recovery transition. Operationally, a missing authenticated slot suspends continuation until its retained witness is recovered; substituting zero is not an authenticated recovery mechanism. A deployment needing explicit missingness must define and version that policy separately. All relation arithmetic is over $F_{p}$. Nonnegative integer interpretation additionally requires the accumulated value and logical counters to remain below *p*; measurements are range-constrained, but the circuit does not impose an independent 64-bit counter limit. This operational no-wrap condition distinguishes field arithmetic from unbounded integer arithmetic.

### 3.4. Architecture Overview

The HeRZ architecture addresses the batch-wide memory growth associated with monolithic proofs by using folding schemes over Relaxed Rank-1 Constraint Systems (Relaxed R1CSs). Rather than generating and verifying a complete proof at each step, the prover incrementally aggregates state-transition instances into a fixed-width accumulator. This design bounds incremental prover memory, although total folding time still grows with the number of events.

**Figure 2:**
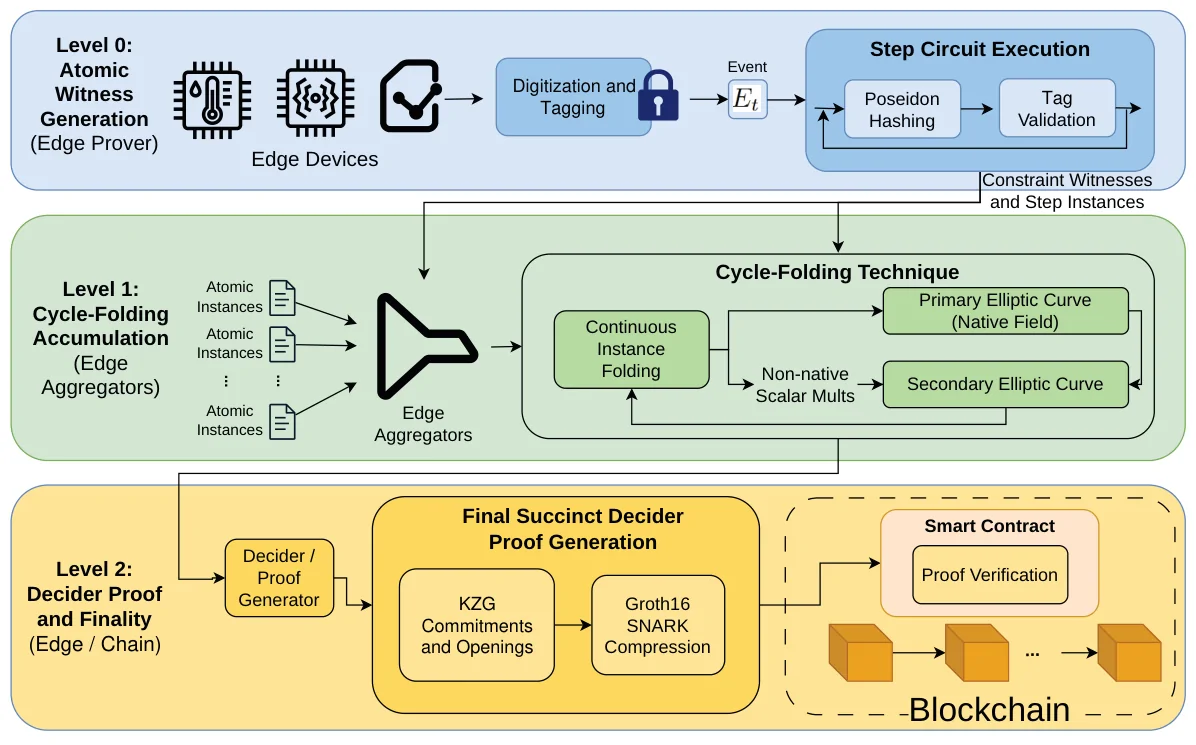
The system architecture of HeRZ. Decider proof generation runs at the secure edge; only verification and state settlement execute on-chain.

As illustrated in Figure 2, the data processing pipeline operates across three hierarchical layers:

1. **Level 0: Event Authentication (Sensor/SE):** Raw signals are digitized into $E_{t}$ and authenticated over the complete event context by the secure element.
2. **Level 1: Step Evaluation and Cycle Folding (Secure Edge Prover):** The per-asset gateway evaluates the witness-dependent step relation and folds the resulting transition into the running accumulator. CycleFold [31] delegates non-native scalar multiplications to the secondary curve. Only folded/public state is released outside the protected proving boundary.
3. **Level 2: Decider Proof, Finality, and Audit (Edge/Chain):** The accumulated instance is compressed into a succinct boundary-to-boundary proof and forwarded, potentially through untrusted relays, to the verifier. A state-advancing invocation atomically replaces the asset’s canonical five-field state; a read-only invocation can instead validate an interval whose boundaries are anchored historical checkpoints.

## 4. Methodology

This section formalizes HeRZ as a verifiable state-evolution pipeline. Phase 1 implements the HeRZ relation for one physical event and state update (Section 4.1); Phase 2 preserves this relation over a sequence of updates through folding (Section 4.2); and Phase 3 settles the folded relation as an EVM-verifiable proof of physical state finality (Section 4.3). Table 3 summarizes the notation used throughout this section.

The methodological core of HeRZ is a cross-domain state-evolution invariant formalized by the relation below. For each private event $E_{t}$ and auxiliary witness $\omega_{t}$, the protocol admits a transition from $z_{t}$ to $z_{t+1}$ only if

(1) $$R_{\mathrm{HeRZ}}\left(z_{t},z_{t+1};E_{t},\omega_{t}\right)={\mathrm{Auth}}_{t}\wedge {\mathrm{Guard}}_{t}\wedge {\mathrm{Update}}_{t}\wedge {\mathrm{Link}}_{t}=1,$$

where ${\mathrm{Auth}}_{t}$ binds the event to the registered device secret, ${\mathrm{Guard}}_{t}$ enforces domain-specific physical predicates, ${\mathrm{Update}}_{t}$ applies the deterministic state transition, and ${\mathrm{Link}}_{t}$ preserves temporal continuity through the history hash. HeRZ lifts this atomic relation to an interval reachability statement:

(2) $$\begin{array}{cc} z_{i}\overset{R_{\mathrm{HeRZ}}^{N_{ij}}}{\to }z_{j}\;⟺\;\; & \exists \;{\left\{\left(E_{t},\omega_{t}\right)\right\}}_{t=i}^{j-1}\;s.t.\\ \; & \overset{j-1}{\underset{t=i}{⋀}}R_{\mathrm{HeRZ}}\left(z_{t},z_{t+1};E_{t},\omega_{t}\right)=1, \end{array}$$

where $N_{ij}=j-i=q_{j}-q_{i}$. The same relation governs all three execution domains: an edge circuit establishes one admissible transition, a folding accumulator preserves the boundary-to-boundary reachability claim, and a succinct verifier checks that claim without observing the intermediate events. If $z_{i}$ is the current canonical state, atomic settlement makes an accepted $z_{j}$ its unique canonical successor. If both endpoints are anchored historical checkpoints, the same statement certifies the selected evolution interval without changing the current state.

**Proposition** **1** (Semantic composition of evolution segments)**.** *If $z_{i}\overset{R_{\mathrm{HeRZ}}^{N_{ij}}}{\to }z_{j}$ and $z_{j}\overset{R_{\mathrm{HeRZ}}^{N_{jk}}}{\to }z_{k}$, then $z_{i}\overset{R_{\mathrm{HeRZ}}^{N_{ij}+N_{jk}}}{\to }z_{k}$.*

The proposition follows by concatenating the witness sequences at their common state $z_{j}$. It is a semantic composition property; producing $\Pi_{\left[i,j\right]}$ still requires the interval’s retained boundaries and private witnesses.

**Figure 3:**
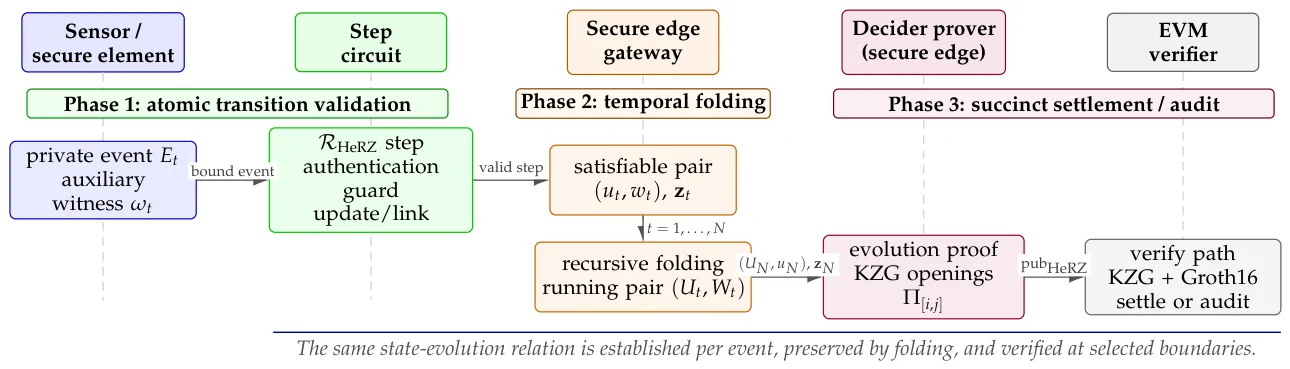
Methodology sequence of HeRZ. The same relation is established per event, preserved between selected boundaries by folding, and verified for canonical settlement or historical interval audit.

Figure 3 summarizes the three-phase execution path: Phase 1 turns one authenticated event into a constrained instance, Phase 2 folds these instances while preserving temporal reachability, and Phase 3 verifies the accumulated relation either as an EVM-settled finality claim or as a read-only certificate for an anchored historical interval.

### 4.1. Phase 1: Atomic Step Validation

Phase 1 begins at the physical boundary and ends with a single satisfiable invocation of the step circuit $C_{\mathrm{step}}$. It is the concrete arithmetic realization of the state-evolution relation for one event: the edge device forms an authenticated physical-event witness, and the circuit checks identity binding, physical guards, deterministic state update, and temporal linkage.

#### 4.1.1. Edge-Side Event and Witness Formation

At this boundary, IoT sensors interface with the digital proving layer. Each edge device is assumed to possess a TEE or secure element (SE) that safeguards a factory-provisioned private secret $sk_{\mathrm{dev}}$. Provisioning assigns an independent secret to each asset, deployment, and key epoch; the protected edge prover is an authorized holder of that secret. The device publishes a public identity anchor computed via Poseidon hashing

(3) $$pk_{\mathrm{dev}}=H_{P}\left(sk_{\mathrm{dev}}\right)$$

This commitment is registered on-chain and carried as a public component of the IVC state. At each discrete time interval *t*, the edge device performs three operations:

1. **Sensor Acquisition.** The device samples raw physical signals, producing a structured event
  (4) $$E_{t}=\left(\Delta_{t},\;V_{t},\;\eta_{t},\;\tau_{t}\right)$$
  where $\Delta_{t}$ denotes the energy generation increment (scaled by $10^{3}$ for finite-field arithmetic compatibility), $V_{t}$ is the measured voltage, $\eta_{t}$ is the binary day/night indicator, and $\tau_{t}$ is the authenticated logical acquisition-slot index.
2. **Cryptographic Binding.** The TEE generates a fresh random salt $r_{t}\overset{\$}{\leftarrow }F_{p}$ and computes a domain-separated authentication tag over the complete event context
  (5) $$\sigma_{t}=H_{P}\left(d_{\mathrm{auth}},sk_{\mathrm{dev}},pk_{\mathrm{dev}},q_{t+1},\Delta_{t},V_{t},\eta_{t},\tau_{t},r_{t}\right)$$
  Here $d_{\mathrm{auth}}$ identifies the event-authentication role. The ordered input binds the complete event, sequence, authenticated logical acquisition-slot index, and salt to the registered device. This acquisition record becomes the authentication input to the step relation. The circuit also checks $pk_{\mathrm{dev}}=H_{P}\left(sk_{\mathrm{dev}}\right)$ against the registered anchor.
3. **Witness Packaging.** The device packages the private auxiliary witness for the step circuit
  (6) $$\omega_{t}=\left(sk_{\mathrm{dev}},\;r_{t},\;\sigma_{t}\right)$$
  Together, $\left(E_{t},\omega_{t}\right)$ supplies the logical private step input. The registered $pk_{\mathrm{dev}}$ comes from the public state; the implementation also allocates an auxiliary copy constrained to equal that public anchor. The physical bounds are protocol constants compiled into the relation rather than prover-selectable witnesses. The secret is consumed only inside the protected edge-proving environment and is never revealed on-chain.

Given $\left(E_{t},\omega_{t}\right)$, each folding step executes one invocation of $C_{\mathrm{step}}$ over the BN254 scalar field. The circuit maps $z_{t}=\left({\mathrm{acc}}_{t},H_{t},pk_{\mathrm{dev}},q_{t},{\bar{\tau }}_{t}\right)\in F_{p}^{5}$ to $z_{t+1}$ while enforcing $q_{t+1}=q_{t}+1$ and $\tau_{t}={\bar{\tau }}_{t}+1$.

#### 4.1.2. ZK-Friendly Poseidon Hash

The Poseidon hash function [32] is used as the sole in-circuit hash primitive because it operates natively over $F_{p}$.

The evaluated configuration is Sonobe’s poseidon_canonical_config at commit 9414c45, instantiated with Arkworks 0.5.0 over ark_bn254::Fr [33,34]. Its documented design target is 120-bit security; the authentication assumption is specified separately in Section 5.2. Table 4 specifies the permutation, sponge interface, and reproducible constant generation.

##### Symmetric Authentication Interface

Let $k=sk_{\mathrm{dev}}$, $a=H_{P}\left(k\right)$, and m=(q,Δ,V,η,τ,r) be an ordered six-field context. The intended MAC interface is

(7) $$\begin{array}{cc} \mathrm{KeyGen}: & \;k\overset{\$}{\leftarrow }F_{p}\setminus \left\{0\right\},\;a\leftarrow H_{P}\left(k\right),\\ {\mathrm{Tag}}_{k}\left(m\right): & \;\sigma \leftarrow H_{P}\left(d_{\mathrm{auth}},k,a,m\right),\\ {\mathrm{Verify}}_{k}\left(m,\sigma \right): & \;[\sigma ={\mathrm{Tag}}_{k}\left(m\right)]. \end{array}$$

The registered anchor *a* is fixed for this interface. In HeRZ, the circuit privately checks both $a=H_{P}\left(k\right)$ and ${\mathrm{Verify}}_{k}=1$, allowing the blockchain to verify the device-bound relation without receiving *k*. This keyed-sponge MAC uses symmetric verification; the public anchor is a key commitment rather than a digital-signature verification key.

##### Key Placement and Domain Separation

The first absorbed rate block is $\left(d_{\mathrm{auth}},k,a,q\right)$: the secret enters the permutation before the remaining public-context fields, following the secret-prefix rationale of keyed sponges [35]. Distinct leading constants separate authentication and history, while the one-field anchor has a separate fixed schema. These fixed arities and field positions make the supported input encodings unambiguous. Section 5.2 analyzes this exact interface, including its public-key-derived anchor. Role separation is complemented by independent keys for asset/deployment isolation (Section 7.2).

#### 4.1.3. Identity-Binding Constraints

The first constraint class establishes cryptographic binding between the physical data and the originating device. HeRZ employs a *symmetric Poseidon-based authentication tag* as defined in Equation (7). The circuit first reconstructs the registered device identity anchor from the private device secret

(8) $${\hat{pk}}_{\mathrm{dev}}=H_{P}\left(sk_{\mathrm{dev}}\right)\overset{!}{=}pk_{\mathrm{dev}}$$

It then computes

(9) $${\hat{\sigma }}_{t}=H_{P}\left(d_{\mathrm{auth}},sk_{\mathrm{dev}},pk_{\mathrm{dev}},q_{t+1},\Delta_{t},V_{t},\eta_{t},\tau_{t},r_{t}\right)$$

and enforces the equality constraint

(10) $${\hat{\sigma }}_{t}\overset{!}{=}\sigma_{t}$$

where $\sigma_{t}$ is the claimed authentication tag provided as a witness input, and $\overset{!}{=}$ denotes an R1CS enforcement (i.e., the constraint system becomes unsatisfiable if violated). The public IVC state carries the same $pk_{\mathrm{dev}}$ value across all steps, and the wrapper contract checks that this anchor has been registered on-chain before accepting the proof.

Thus, a satisfying step opens the registered anchor and reproduces the acquisition tag using the same secret and event fields. Those fields are also consumed by the physical guards, deterministic update, and history commitment. This shared-variable binding carries the authenticated acquisition record through the entire transition, under the trust model in Section 3.1.

Algorithm 1 provides an integrated view of the complete step relation; its physical-guard and deterministic-update components are detailed next.

#### 4.1.4. Physical-Guard Constraints

The second constraint layer transforms domain-specific physical rules into arithmetic predicates. We define four guard sub-constraints:

The nonnegative energy increment must satisfy $0\leq \Delta_{t}\leq \Delta_{\max}$, and voltage must satisfy $V_{t}\leq V_{\max}$. The implementation allocates internal upper-bound operands and constrains them equal to the compiled policy constants before applying the range gadgets; hence a prover cannot relax the policy by choosing alternative thresholds. In the evaluated PV configuration, generation readings are encoded as $\Delta_{t}=\mathrm{round}\left(10^{3}\Delta_{t}^{\mathrm{kWh}}\right)$, voltage is represented in millivolts, and the circuit compiles $\Delta_{\max}=600,000$ (600 kWh per hourly slot) and $V_{\max}=250,000$ mV (250 V). The context bit is set to $\eta_{t}=1$ for local hours 20:00–05:59 and to zero otherwise. HeRZ then enforces $\eta_{t}\Delta_{t}=0$ and $\eta_{t}\left(1-\eta_{t}\right)=0$. These values specify the evaluated policy configuration rather than universal PV limits.
**Algorithm 1** HeRZ atomic state-transition circuit ($C_{\mathrm{step}}$)**Require:** IVC state              $z_{t}=\left({\mathrm{acc}}_{t},H_{t},pk_{\mathrm{dev}},q_{t},{\bar{\tau }}_{t}\right)$;              private event $E_{t}=\left(\Delta_{t},V_{t},\eta_{t},\tau_{t}\right)$;              auxiliary witness $\omega_{t}=\left(sk_{\mathrm{dev}},r_{t},\sigma_{t}\right)$**Ensure:**   Next state              $z_{t+1}=\left({\mathrm{acc}}_{t+1},H_{t+1},pk_{\mathrm{dev}},q_{t+1},{\bar{\tau }}_{t+1}\right)$**// Identity-binding constraints**  1:${\hat{pk}}_{\mathrm{dev}}\leftarrow H_{P}\left(sk_{\mathrm{dev}}\right)$  2:**enforce** ${\hat{pk}}_{\mathrm{dev}}=pk_{\mathrm{dev}}\;$▹ registered identity anchor  3:$q_{t+1}\leftarrow q_{t}+1$; **enforce** $\tau_{t}={\bar{\tau }}_{t}+1$  4:${\hat{\sigma }}_{t}\leftarrow H_{P}\left(d_{\mathrm{auth}},sk_{\mathrm{dev}},pk_{\mathrm{dev}},q_{t+1},\Delta_{t},V_{t},\eta_{t},\tau_{t},r_{t}\right)$  5:**enforce** ${\hat{\sigma }}_{t}=\sigma_{t}\;$▹ bind complete event and order**// Physical-guard constraints**  6:allocate ${\hat{\Delta }}_{\max},{\hat{V}}_{\max}$ and constrain them to protocol constants  7:**enforce** $0\leq \Delta_{t}\leq {\hat{\Delta }}_{\max}\;$▹$C_{1}$: fixed range policy  8:**enforce** $V_{t}\leq {\hat{V}}_{\max}\;$▹$C_{2}$: fixed voltage policy  9:**enforce** $\eta_{t}\cdot \Delta_{t}=0\;$▹$C_{3}$: quadratic night constraint10:**enforce** $\eta_{t}\cdot \left(1-\eta_{t}\right)=0\;$▹$C_{4}$: boolean constraint**// Deterministic state update**11:${\mathrm{acc}}_{t+1}\leftarrow {\mathrm{acc}}_{t}+\Delta_{t}\;$▹$S_{1}$: homomorphic accumulation12:$H_{t+1}\leftarrow H_{P}\left(d_{\mathrm{hist}},H_{t},pk_{\mathrm{dev}},q_{t+1},\Delta_{t},V_{t},\eta_{t},\tau_{t},r_{t},\sigma_{t}\right)$13:${\bar{\tau }}_{t+1}\leftarrow \tau_{t}$14:**return** $z_{t+1}=\left({\mathrm{acc}}_{t+1},H_{t+1},pk_{\mathrm{dev}},q_{t+1},{\bar{\tau }}_{t+1}\right)$

The complete set of physical guards can be expressed as the conjunction

(11) $$\begin{array}{cc} \mathrm{Guard}(E_{t})= & \underset{C_{1}:\;\mathrm{range}}{\underset{︸}{\left(0\leq \Delta_{t}\leq \Delta_{\max}\right)}}\;\wedge \;\underset{C_{2}:\;\mathrm{voltage}}{\underset{︸}{\left(V_{t}\leq V_{\max}\right)}}\\ & \;\wedge \;\underset{C_{3}:\;\mathrm{context}}{\underset{︸}{\left(\eta_{t}\cdot \Delta_{t}=0\right)}}\;\wedge \;\underset{C_{4}:\;\mathrm{boolean}}{\underset{︸}{\left(\eta_{t}\in \left\{0,1\right\}\right)}} \end{array}$$

#### 4.1.5. State Machine Update

Only after identity binding and physical guards are satisfied does the circuit perform the deterministic transition. The state vector $z=\left(\mathrm{acc},H,pk_{\mathrm{dev}},q,\bar{\tau }\right)\in F_{p}^{5}$ evolves according to

##### S1: Homomorphic Accumulation


(12) $${\mathrm{acc}}_{t+1}={\mathrm{acc}}_{t}+\Delta_{t}$$

This linear constraint adds a single R1CS gate. Its fixed-width additive form lets the public state carry the cumulative value while each event is evaluated exactly once before its constrained instance is folded.

##### S2: Cryptographic History Hash Chain


(13) $$H_{t+1}=H_{P}\left(d_{\mathrm{hist}},H_{t},pk_{\mathrm{dev}},q_{t+1},\Delta_{t},V_{t},\eta_{t},\tau_{t},r_{t},\sigma_{t}\right)$$

The distinct domain tag $d_{\mathrm{hist}}$ separates history commitments from authentication tags and binds every authenticated transition field to the event order.

### 4.2. Phase 2: Recursive Temporal Aggregation

The core scalability contribution of HeRZ lies in compressing an arbitrarily long sequence of *N* validated state transitions into a single accumulator while preserving the sequential semantics of $R_{\mathrm{HeRZ}}$. The aggregation problem is therefore not merely to batch many circuit executions but to maintain the temporal invariant

(14) $$\begin{array}{cc} I_{t}: & z_{t}=F_{t}\left(z_{t-1},E_{t},\omega_{t}\right),\\ \; & R_{\mathrm{HeRZ}}\left(z_{t-1},z_{t};E_{t},\omega_{t}\right)=1,\;t=1,\ldots ,N, \end{array}$$

inside a compact proof object. HeRZ realizes this invariant with folding-based IVC: each valid step contributes one constrained instance, and the accumulator carries the algebraic obligation that all previously folded transitions remain satisfiable.

#### 4.2.1. IVC Formalization

For a state transition function $F_{t}$ induced by $C_{\mathrm{step}}$, an IVC proof at time *t* should certify the indexed claim

(15) $$\begin{array}{cc} \; & \exists \;{\left\{\left(E_{j},\omega_{j}\right)\right\}}_{j=1}^{t}\;s.t.\\ \; & z_{j}=F_{j}\left(z_{j-1},E_{j},\omega_{j}\right),\\ \; & R_{\mathrm{HeRZ}}\left(z_{j-1},z_{j};E_{j},\omega_{j}\right)=1,\;\forall j\leq t. \end{array}$$

In HeRZ, the public state dimension is fixed at k=5, with $z_{t}=\left({\mathrm{acc}}_{t},H_{t},pk_{\mathrm{dev}},q_{t},{\bar{\tau }}_{t}\right)$. For a genesis state, ${\mathrm{acc}}_{0}=H_{0}=q_{0}={\bar{\tau }}_{0}=0$; subsequent batches start from the asset’s state already stored by the wrapper. Equation (15) is the formal object that the folding layer preserves: the blockchain does not re-execute each $F_{j}$, but it verifies a compressed certificate that the final state is reachable only through a sequence of valid authenticated, ordered, and policy-compliant transitions.

#### 4.2.2. Nova Folding Scheme

Nova achieves IVC through a *folding scheme* that algebraically combines two R1CS instances into one, rather than recursively verifying SNARK proofs inside circuits. This avoids the prohibitive cost of in-circuit proof verification.

##### R1CS and Relaxed R1CS

For public inputs $x\in F_{p}^{n_{x}}$ and private witness $w\in F_{p}^{n_{w}}$, a standard R1CS with matrices $A,B,C\in F_{p}^{m\times (1+n_{x}+n_{w})}$ requires

(16) (A·Z)∘(B·Z)=C·Z

Here, Z=(1,x,w) and ∘ denotes the Hadamard (element-wise) product.

Nova introduces *Relaxed R1CS*, augmenting the system with a scalar $u\in F_{p}$ and an error vector $E\in F_{p}^{m}$:

(17) (A·Z)∘(B·Z)=u·(C·Z)+E

For the relaxed relation, Z=(u,x,w) replaces the leading 1 by *u*; u=1 and E=0 recover standard R1CS. The relaxation is essential for enabling algebraic folding.

##### Committed Relaxed R1CS Instance

A committed Relaxed R1CS public instance $U=(\bar{E},u,\bar{W},x)$ uses Pedersen or KZG [36] commitments $\bar{E}=\mathrm{Commit}\left(E\right)$ and $\bar{W}=\mathrm{Commit}\left(w\right)$. It is satisfied by witness data *W* that open these commitments to E,w and satisfy (17).

##### Folding Protocol

Fold the running pair $\left(U_{1},W_{1}\right)$ with the incoming standard pair $\left(U_{2},W_{2}\right)$, whose scalar $u_{2}=1$ and error $E_{2}=0$:

1. The prover computes the *cross-term* vector
  (18) $$T=AZ_{1}\circ BZ_{2}+AZ_{2}\circ BZ_{1}-u_{1}\cdot CZ_{2}-u_{2}\cdot CZ_{1}$$
  and commits it as $\bar{T}=\mathrm{Commit}\left(T\right)$.
2. After committing to T, derive the transcript challenge
  (19) $$\rho =H_{P}\left(U_{1},U_{2},\bar{T}\right)$$
3. The folded public instance is computed as
  (20) $$\begin{array}{cc} {\bar{E}}^{\prime } & ={\bar{E}}_{1}+\rho \cdot \bar{T}+\rho^{2}\cdot {\bar{E}}_{2} \end{array}$$
  (21) $$\begin{array}{cc} u^{\prime } & =u_{1}+\rho \cdot u_{2} \end{array}$$
  (22) $$\begin{array}{cc} {\bar{W}}^{\prime } & ={\bar{W}}_{1}+\rho \cdot {\bar{W}}_{2} \end{array}$$
  (23) $$\begin{array}{cc} x^{\prime } & =x_{1}+\rho \cdot x_{2} \end{array}$$

**Theorem** **1** (Folding Soundness [21])**.** *For fixed matrices and commitment parameters, bind $U_{1},U_{2}$ and $\bar{T}$ before deriving ρ. Under commitment binding and the folding scheme’s Fiat–Shamir assumption, producing a satisfying pair $\left(U^{\prime },W^{\prime }\right)$, with $U^{\prime }=\left({\bar{E}}^{\prime },u^{\prime },{\bar{W}}^{\prime },x^{\prime }\right)$, while either input instance is unsatisfiable has negligible probability for an efficient prover.*

This theorem is the cryptographic foundation that enables HeRZ to compress *N* step validations into a single Relaxed R1CS instance without loss of soundness.

#### 4.2.3. CycleFold: Efficient Non-Native Arithmetic

A critical implementation challenge arises from the fact that the commitment scheme operates over elliptic curve groups, where scalar multiplications involve non-native field arithmetic when verified inside the circuit. CycleFold [31] addresses this by delegating the scalar multiplication verification to a secondary elliptic curve.

HeRZ instantiates the cycle of curves as follows:

- **Primary curve:** BN254 ($G_{1}$), whose scalar field $F_{p}$ hosts the step circuit $C_{\mathrm{step}}$.
- **Secondary curve:** Grumpkin, whose base field equals the BN254 scalar field, enabling efficient cross-curve verification.

##### Curve Parameters and Effective Security

The curve parameters and generators are fixed by Arkworks 0.5.0 (ark-bn254 and ark-grumpkin) [37,38]. Writing *p* for the step-field prime in Table 4, BN254 is $y^{2}=x^{3}+3$ over $F_{\ell }$ with group order *p*, whereas Grumpkin is $y^{2}=x^{3}-17$ over $F_{p}$ with group order *ℓ*, where

ℓ=21888242871839275222246405745257275088696311157297823662689037894645226208583.

The reciprocal base/scalar fields support CycleFold, while BN254 supports the EVM pairing checks used by KZG and Groth16. These library parameters are fixed independently of the dataset and event count.

The two approximately 254-bit orders give comparable generic discrete-logarithm work factors of roughly $2^{127}$. Their effective security is nevertheless unequal: BN254 also admits a pairing reduction to its embedding-degree-12 target field, where number-field-sieve estimates for 254-bit BN curves are around 100 bits, depending on the attack cost model [39]. Grumpkin’s generic group estimate therefore does not raise the security of the BN254-based commitment and Decider layers. HeRZ uses this cycle for recursive/EVM compatibility; it does not claim uniform 128-bit security. The Poseidon configuration’s 120-bit target is a separate component parameter.

For the primary curve, HeRZ uses KZG [36] as the commitment layer for the folded witness and relaxation error. This choice gives the off-chain accumulator an on-chain verification semantics: the EVM verifier only needs to check that the committed vectors open consistently at transcript-derived challenge points, rather than inspect the vectors themselves. Formally, a witness vector is committed as

(24) $$\mathrm{KZG}.\mathrm{Commit}\left(w\right)=\sum_{i=0}^{n-1}w_{i}\cdot {\left[\tau^{i}\right]}_{1}$$

where ${\left[\tau^{i}\right]}_{1}$ are the Structured Reference String (SRS) elements from a trusted setup ceremony, and τ is the secret trapdoor. The resulting commitment is binding under the standard KZG assumption, while its pairing-based opening check matches the BN254 precompiles used in Phase 3. Thus, KZG is not treated as a black-box replacement for Pedersen; it is the bridge that makes HeRZ’s folded state relation economically verifiable by an EVM contract. The soundness claim therefore relies on the Nova folding argument instantiated with a binding polynomial commitment, a correctly generated SRS, and Fiat–Shamir challenges that bind the folded instances, cross-term commitment, and public state transcript. The evaluated implementation uses Sonobe’s non-hiding KZG mode. Consequently, the KZG layer is not claimed to conceal committed vectors: its commitments and challenge-point evaluations may reveal algebraic information. Groth16 protects the Decider witness that is not otherwise exposed, whereas transcript-level leakage analysis and a hiding polynomial-commitment instantiation are outside the present guarantees.

#### 4.2.4. Incremental Folding Loop

The HeRZ folding loop iterates over *N* time steps. Let the batch’s initial state be

(25) $$\left(U_{0},\;W_{0},\;z_{0}\right)=\left(U_{\mathrm{init}},\;W_{\mathrm{init}},\;\left({\mathrm{acc}}_{0},H_{0},pk_{\mathrm{dev}},q_{0},{\bar{\tau }}_{0}\right)\right)$$

where $U_{\mathrm{init}}$ and $W_{\mathrm{init}}$ denote the initial accumulated Relaxed R1CS instance and its corresponding witness state. For each step t=1,…,N, the edge event induces a fresh constrained instance $I_{t}$ for the relation in (1). Folding then applies a challenge-dependent aggregation map:

(26) $$\begin{array}{cc} I_{t}=\left(u_{t},w_{t}\right) & \leftarrow R1\mathrm{CS}\left(C_{\mathrm{step}},z_{t-1},E_{t},\omega_{t},z_{t}\right),\\ \left(U_{t},W_{t}\right) & \leftarrow {\mathrm{Fold}}_{\rho_{t}}\left(\left(U_{t-1},W_{t-1}\right),I_{t}\right), \end{array}$$

where $\rho_{t}$ is transcript-derived and $I_{t}$ is satisfiable only when the step relation accepts. Here, $w_{t}$ is the R1CS witness paired with instance $u_{t}$, whereas $\omega_{t}$ is the BCPS auxiliary witness used to synthesize that pair. The concrete Nova/Sonobe interface retains the running pair $\left(U_{N},W_{N}\right)$ and the incoming pair $\left(u_{N},w_{N}\right)$; the Decider checks their final challenge-dependent fold. Thus, public instances $\left(U_{N},u_{N}\right)$, final state $z_{N}$, and the committed private witnesses encode the *N* validated transitions.

At each iteration, the prover:

1. Synthesizes the step circuit $C_{\mathrm{step}}$ with $\left(E_{t},\omega_{t}\right)$, producing an incoming R1CS pair $I_{t}=\left(u_{t},w_{t}\right)$.
2. Folds $\left(U_{t-1},W_{t-1}\right)$ with $\left(u_{t},w_{t}\right)$ using the protocol of Section 4.2.2, yielding $\left(U_{t},W_{t}\right)$.

This recurrence gives HeRZ its academic object of proof: the accumulator is a compressed certificate of reachability in the physical-state machine, not only a compressed record of many circuit executions.

**Proposition** **2** (Constant Memory)**.** *The memory footprint of the Nova accumulator $\left(U_{t},W_{t}\right)$ is $O(|C_{\mathrm{step}}|)$ regardless of the number of folded steps N. This is in contrast to monolithic batch approaches where the circuit size grows as $O(N\cdot |C_{\mathrm{step}}|)$.*

After processing all *N* events, the Decider input comprises the step count *N*, boundary states $\left(z_{0},z_{N}\right)$, running relaxed pair $\left(U_{N},W_{N}\right)$, and incoming pair $\left(u_{N},w_{N}\right)$. Here, $z_{N}=\left({\mathrm{acc}}_{N},H_{N},pk_{\mathrm{dev}},q_{0}+N,{\bar{\tau }}_{N}\right)$. Only $U_{N}$ and $u_{N}$ are public instance data; $W_{N}$ and $w_{N}$ remain private and are linked through polynomial commitments and openings.

### 4.3. Phase 3: Deferred Verification, Canonical Finality, and Historical Audit

After *N* step evaluations, public Nova instances $\left(U_{N},u_{N}\right)$ and their committed private witnesses encode that every event $E_{1},\ldots ,E_{N}$ is device-bound, policy-compliant, and consistently incorporated into $z_{N}$. Phase 3 interprets this accumulator as a reachability claim: the final boundary is reachable from the initial boundary through relation-valid transitions. The subscripts 0 and *N* are local to the selected interval and need not denote genesis and the latest lifecycle state. Event processing and folding occur off-chain, while one succinct verification either advances the canonical state or audits an anchored historical segment with verification cost independent of *N*.

#### 4.3.1. HeRZ Physical State Commitment

Before describing the compression mechanism, we formalize the semantics of on-chain finality. When the verifier smart contract accepts a HeRZ proof, it establishes the following Physical State Commitment:

**Definition** **2** (HeRZ Physical State Commitment)**.** *A HeRZ proof $\Pi_{\mathrm{HeRZ}}$ for the tuple $\left(N,z_{0},z_{N}\right)$ constitutes a Physical State Commitment if and only if the on-chain verifier confirms that there exist N events $E_{1},\ldots ,E_{N}$ such that:*

*1.*  ***Device and Event Authenticity:*** *Each $E_{t}$ and its order were bound to a registered anchor $pk_{\mathrm{dev}}=H_{P}\left(sk_{\mathrm{dev}}\right)$ through the domain-separated tag in (5).*
*2.*  ***Physical-Policy Compliance:*** *Each $E_{t}$ satisfied all guard predicates $Guard(E_{t})=true$.*
*3.*  ***State Integrity and Continuity:*** *The final state $z_{N}=\left({\mathrm{acc}}_{N},H_{N},pk_{\mathrm{dev}},q_{N},{\bar{\tau }}_{N}\right)$ is the unique output of the deterministic transition chain, with $q_{N}=q_{0}+N$, strictly advancing logical slots, and an unchanged device anchor.*

Here, “physical” identifies the asset-state application and its encoded policy, not independent attestation of environmental truth. “Finality” denotes consensus-finalized acceptance of the relation and atomic canonical-state advancement. The PSC therefore certifies a device-bound, policy-compliant evolution path; correspondence of its measurements to the environment follows the acquisition assumptions in Section 3.1.

#### 4.3.2. Dual Verification Semantics

The proof statement is boundary-parametric. For any retained interval [i,j] with $N_{ij}=j-i\geq 2$, the prover can initialize the same step circuit with $z_{i}$, process the private events and witnesses through $z_{j}$, and produce

(27) $$\Pi_{\left[i,j\right]}\leftarrow \mathrm{Prove}\left(N_{ij},z_{i},z_{j};{\left\{E_{t},\omega_{t}\right\}}_{t=i}^{j-1}\right).$$

If $z_{i}$ equals the wrapper’s current state, verification atomically installs $z_{j}$ as the unique canonical successor. If both endpoints match previously anchored checkpoints, the same proof can instead certify the interval read-only without exposing its events or changing canonical state. Read-only audits require both endpoints to be anchored checkpoints within the same protocol version and the corresponding interval witnesses to be available from the authorized archive.

#### 4.3.3. HeRZ Commitment Scheme: EVM-Native KZG

A key HeRZ design decision is the choice of commitment scheme for the primary folding curve. While the default Nova construction uses Pedersen commitments (which require O(n) scalar multiplications for verification), HeRZ instantiates the commitment interface with KZG [36] polynomial commitments for the BN254 primary curve. This deployment-specific commitment layer aligns the Decider with the EVM’s native BN254 pairing support while preserving the same relaxed-R1CS folding relation. KZG changes how the witness, error vector, and cross-term commitments are bound and opened to the Decider; the state-evolution semantics remain enforced by the relation and Decider circuit.

The EVM provides native precompiled contracts for BN254 pairing operations at addresses 0x06 (point addition, 150 gas), 0x07 (scalar multiplication, 6000 gas), and 0x08 (pairing check, ∼113,000 gas). While these primitive costs explain the verifier cost model, they do not directly yield an end-to-end gas measurement. By adopting KZG, HeRZ reduces each commitment opening verification to a single pairing equation

(28) $$e\left(\pi ,{\left[\tau \right]}_{2}-x\cdot G_{2}\right)=e\left(C-y\cdot G_{1},G_{2}\right)$$

where $e:G_{1}\times G_{2}\to G_{T}$ is the bilinear pairing, π is the KZG opening proof, *C* is the polynomial commitment, *x* is the evaluation point (challenge), and *y* is the claimed evaluation.

For gas optimization, HeRZ reformulates this equation to avoid $G_{2}$ scalar multiplications entirely, leveraging the EVM’s cheaper $G_{1}$ operations

(29) $$e\left(\pi ,\mathrm{VK}\right)\cdot e\left(-x\cdot \pi -C+y\cdot G_{1},G_{2}\right)=1$$

This reformulation ensures that HeRZ’s on-chain verification uses only $G_{1}$ scalar multiplications (6000 gas each) rather than the prohibitively expensive $G_{2}$ counterparts, lowering the modeled cost of each KZG opening inside the verifier.

#### 4.3.4. HeRZ Proof Compression via Groth16

After *N* folding steps, the HeRZ accumulator contains the full Relaxed R1CS state, which cannot be directly submitted on-chain because the witness is private and raw verification costs $O(|C_{\mathrm{step}}|)$. HeRZ addresses this through a proof compression step: it applies a Groth16 [40] proving system to generate a constant-size SNARK that attests to the correctness of the entire folding history.

HeRZ constructs an *augmented Decider circuit* $C_{\mathrm{dec}}$ that verifies the Nova folding relation, the consistency of the accumulated Relaxed R1CS instance, and the CycleFold cross-curve checks. The circuit size is independent of the number of folded steps *N*, although it is larger than the step circuit. Thus, constant-size verification should not be read as constant-cost proving; folding and final Decider proving still occur off-chain. The Groth16 prover compresses this fixed Decider circuit into $\pi_{\mathrm{HeRZ}}=\left(\pi_{A},\pi_{B},\pi_{C}\right)\in G_{1}\times G_{2}\times G_{1}$, whose size is independent of *N*. The serialized HeRZ Decider object additionally carries two KZG opening records (including their evaluations), the final cross-term commitment $\bar{T}$, the folding challenge ρ, and the two opening challenges $\chi_{W},\chi_{E}$. Accordingly,

(30) $$\Pi_{\mathrm{HeRZ}}=\left(\pi_{\mathrm{HeRZ}},\pi_{W}^{\mathrm{KZG}},v_{W},\pi_{E}^{\mathrm{KZG}},v_{E},\bar{T},\rho ,\chi_{W},\chi_{E}\right),$$

where the concrete encoding follows the Sonobe Decider proof structure. The constraint counts and serialized object size are reported in Section 6.

The public inputs to the HeRZ Decider circuit encode the complete RWA attestation. In the notation below, $\left(z_{0},z_{N}\right)$ are the local boundaries of the selected interval:

(31) $$\begin{array}{cc} {\mathrm{pub}}_{\mathrm{HeRZ}}= & (\underset{\mathrm{HeRZ}\;\mathrm{state}\;\mathrm{attestation}}{\underset{︸}{H\left(\mathrm{vp}\right),\;N,\;z_{0},\;z_{N}}},\;\underset{\mathrm{accumulator}\;\mathrm{commitments}}{\underset{︸}{\mathrm{limbs}\left(\bar{W}\right),\;\mathrm{limbs}\left(\bar{E}\right)}},\\ \; & \;\;\underset{\mathrm{KZG}\;\text{challenges/evals}}{\underset{︸}{\chi_{W},\;\chi_{E},\;v_{W},\;v_{E}}},\;\underset{\text{cross-term}}{\underset{︸}{\mathrm{limbs}(\bar{T})}}). \end{array}$$

Here, the  state attestation fields represent physical quantities:

- *N*: number of verified measurement periods.
- $z_{0}=($${\mathrm{acc}}_{0}$$,H_{0},pk_{\mathrm{dev}},q_{0},{\bar{\tau }}_{0})$: canonical asset state at the start of this settlement batch.
- $z_{N}=($${\mathrm{acc}}_{N}$$,H_{N},pk_{\mathrm{dev}},q_{0}+N,{\bar{\tau }}_{N})$: final state, encoding cumulative output, tamper-evident history, unchanged identity, and advanced sequence/time.
- H(vp): commitment to additional verification parameters (e.g., circuit-specific public parameters).

The remaining fields are cryptographic inputs used to verify the Nova accumulator.

The limbs(·) function decomposes elliptic curve point coordinates into 5 limbs of 55 bits each, ensuring compatibility with the BN254 scalar field:

(32) $$\mathrm{limbs}\left(x\right)=\left(x_{0},x_{1},x_{2},x_{3},x_{4}\right),\;x=\sum_{i=0}^{4}x_{i}\cdot 2^{55i}.$$

#### 4.3.5. HeRZ On-Chain Verifier Architecture

HeRZ generates a Solidity verifier contract that serves as the on-chain trust anchor. The contract implements three distinct verification layers:

1. **KZG10Verifier**: Checks the claimed evaluations of the combined witness and error commitments $\left({\bar{W}}^{\prime },{\bar{E}}^{\prime }\right)$ using the optimized pairing equation in Equation (29). This layer establishes opening consistency; the Decider circuit supplies the relation semantics.
2. **Groth16Verifier**: Verifies the fixed Decider circuit, which certifies the folded relation, accumulator consistency, and public-state linkage without re-executing all *N* step circuits on-chain. The verification-key constants (α, β, γ, δ, $\left\{IC_{i}\right\}$) are hard-coded and circuit-specific.
3. **HeRZDecider**: Orchestrates the two sub-verifiers and reconstructs the combined commitments from the folding randomness ρ, thereby ensuring end-to-end soundness of the Physical State Commitment (Definition 2).

Algorithm 2 summarizes the complete on-chain verification procedure.

Upon successful verification, the smart contract stores and emits $z_{N}=\left({\mathrm{acc}}_{N},H_{N},pk_{\mathrm{dev}},q_{N},{\bar{\tau }}_{N}\right)$. Downstream RWA contracts may consume this verified state as an oracle input: ${\mathrm{acc}}_{N}$ can feed application-specific minting or distribution rules, $H_{N}$ commits the event history, and $\left(q_{N},{\bar{\tau }}_{N}\right)$ prevent a later batch from reusing an earlier state.
**Algorithm 2** HeRZ on-chain physical state verification**Require:**           Folded states $\left(N,z_{0},z_{N}\right)$;           accumulator commitments; folding challenge ρ;           KZG challenges/evaluations;           opening proofs $\left(\pi_{W}^{\mathrm{KZG}},\pi_{E}^{\mathrm{KZG}}\right)$;           and Decider proof $\pi_{\mathrm{HeRZ}}$**Ensure:** Accept $z_{N}$ or revert  1:**require** N≥2 and $pk_{\mathrm{dev}}$ is registered and active  2:**require** $z_{0}\left[2\right]=z_{N}\left[2\right]=pk_{\mathrm{dev}}$  3:**require** canonical field encodings of $N,z_{0},z_{N}$  4:**require** $q_{N}-q_{0}={\bar{\tau }}_{N}-{\bar{\tau }}_{0}=N$ without integer wraparound  5:**require** $z_{0}=\mathrm{canonicalState}\left[pk_{\mathrm{dev}}\right]$  6:${\bar{W}}^{\prime }\leftarrow {\bar{W}}_{U}+\rho \;{\bar{W}}_{u}$  7:${\bar{E}}^{\prime }\leftarrow {\bar{E}}_{U}+\rho \;\bar{T}$  8:**require** $\mathrm{KZG}.\mathrm{check}({\bar{W}}^{\prime },\chi_{W},v_{W},\pi_{W}^{\mathrm{KZG}})$  9:**require** $\mathrm{KZG}.\mathrm{check}({\bar{E}}^{\prime },\chi_{E},v_{E},\pi_{E}^{\mathrm{KZG}})$10:pub←buildHeRZInputs(fieldsinEquation(31))11:**require** $\mathrm{Groth16}.\mathrm{verify}(\pi_{\mathrm{HeRZ}},\mathrm{pub})$12:$\mathrm{canonicalState}\left[pk_{\mathrm{dev}}\right]\leftarrow z_{N}$13:Store $\mathrm{Digest}(v,z_{N})$ at $\mathrm{registry}[pk_{\mathrm{dev}},q_{N}]$14:**return** $z_{N}$

Algorithm 2 atomically advances the canonical asset state. Registration anchors the initialized state, and every successful settlement additionally stores a digest of its endpoint under the deployment version and device sequence. These are the only checkpoint-registration paths; the owner cannot insert arbitrary historical endpoints. The wrapper version is derived from its chain, address, and immutable verifier address. The registry stores one 32-byte digest per checkpoint; transaction logs expose the corresponding boundary state.

#### 4.3.6. Read-Only Historical Audit

Algorithm 3 gives the complementary path. A request selects any retained interval whose two endpoints are already anchored in the same version; it does not select arbitrary unanchored event positions. A requested non-checkpoint boundary must be reported as unavailable rather than silently rounded to another period. The prover initializes a fresh fold at $z_{i}$ and replays the retained events to $z_{j}$. It does not extract a subproof from a previously settled proof, and the cost of reconstruction grows with j−i.

The protected archive retains the ordered event fields, salts, tags, required key material, and boundary states together with their version. Compaction to a final proof alone is insufficient: that proof does not recover private intermediate witnesses. Before proving, reconstruction checks witness availability and recomputes the terminal state. Missing or inconsistent witnesses produce an explicit error and no proof. On-chain verification authenticates both full boundary states against their registry digests and uses the same Decider verification as settlement. The function is view; it neither checks that the start is the latest state nor writes canonical state, registry entries, or events. Repeating a historical query is allowed. Device revocation stops new settlements but does not invalidate already anchored history.

Audit consumers read the checkpoint registry at a consensus-finalized block under the deployment’s chain policy. The local EVM tests validate contract behavior, not the consensus finality of a live network. Thus, endpoint authentication is a concrete registry check, while checkpoint finality retains the same ledger assumption as forward settlement.
**Algorithm 3** Same-version historical interval audit**Require:** Version *v*; anchored candidates $z_{i},z_{j}$; retained witnesses**Ensure:** A read-only audit result or an explicit failure  1:**require** v=wrapper.version and $N=q_{j}-q_{i}\geq 2$  2:**require** both endpoints carry the same registered device anchor  3:**require** $\mathrm{registry}\left[pk,q_{i}\right]=\mathrm{Digest}\left(v,z_{i}\right)$  4:**require** $\mathrm{registry}\left[pk,q_{j}\right]=\mathrm{Digest}\left(v,z_{j}\right)$  5:Retrieve the *N* consecutive retained event/witness pairs  6:**if** any pair is unavailable **then**  7:    **return** MissingWitness  8:**end if**  9:Recompute the interval from $z_{i}$ and check its endpoint is $z_{j}$10:**if** reconstruction fails **then**11:    **return** InvalidRetainedWitness12:**end if**13:Initialize fresh folding at $z_{i}$; fold the *N* reconstructed transitions14:Generate $\Pi_{i,j}$ with public boundaries $\left(N,z_{i},z_{j}\right)$15:Call verifyHistoricalProof$\left(v,\Pi_{i,j}\right)$   The contract repeats lines 1–4 and validates the interval counters   It checks both KZG openings and the Groth16 Decider proof16:**return** acceptance; **do not modify** canonical state or checkpoints

## 5. Security Analysis

### 5.1. Adversary Model and Security Goal

We consider a probabilistic polynomial-time adversary that controls the untrusted relay/fog service and the communication path to the blockchain. The adversary observes all public settlement data, may obtain previously authenticated events and valid proofs, and may alter, omit, delay, reorder, replay, or combine submitted messages. It may also submit arbitrary verifier calldata and attempt to settle a competing successor state. The adversary does not extract $sk_{\mathrm{dev}}$ from the secure hardware boundary, modify the registered circuit or verification key, or compromise blockchain consensus and contract execution. Availability attacks are permitted but do not constitute an integrity break.

**Definition** **3** (Relation reachability)**.** *For a registered device anchor $pk_{\mathrm{dev}}$, a state $z_{j}$ is $R_{\mathrm{HeRZ}}$-reachable from $z_{i}$ in $N_{ij}$ steps if both states carry the same anchor, $N_{ij}=q_{j}-q_{i}$, and there exist events and auxiliary witnesses ${\left\{\left(E_{t},\omega_{t}\right)\right\}}_{t=i}^{j-1}$ satisfying *(2)*.*

**Definition** **4** (Anchored checkpoint and VSEI)**.** *An anchored checkpoint is the initialized state recorded by finalized registration or an endpoint recorded by a finalized, accepted HeRZ settlement, authenticated in the corresponding version’s checkpoint registry. VSEI requires two properties. First, every proof accepted by the HeRZ proof verifier must establish relation reachability between its public boundary states. Second, every state-advancing settlement must start from the contract’s current canonical state and atomically make its reachable endpoint the unique canonical predecessor consumed by the next settlement. A retrospective interval audit is anchored when both of its public boundary states are anchored checkpoints.*

The security argument uses the following explicit assumptions:

**A1.**  For an uncompromised key and its public anchor, the authentication family in Equation (7) satisfies the auxiliary-anchor PRF assumption of Section 5.2. The anchor is preimage- and second-preimage-resistant, and the history hash is collision-resistant. Event origination additionally relies on the trusted acquisition/proving boundary and the independent-key provisioning policy.
**A2.**  The KZG commitment layer is binding under its generated SRS, and Fiat–Shamir challenges bind the public state, incoming instance, running instance, and cross-term commitment.
**A3.**  The instantiated Nova/CycleFold folding argument and Groth16 Decider are computationally sound for the generated circuits and verification keys.
**A4.**  The EVM atomically executes Algorithm 2. Finalized contract state follows the blockchain’s stated finality model.

**Theorem** **2** (Path Soundness and Canonical VSEI)**.** *Under assumptions **A2**–**A3**, every proof accepted by the HeRZ proof verifier for $\left(N_{ij},z_{i},z_{j}\right)$ establishes that $z_{j}$ is $R_{\mathrm{HeRZ}}$-reachable from $z_{i}$, except with negligible probability. Under **A1**, the events are additionally bound to the registered device’s trusted acquisition domain as specified in Section 3.1 and Theorem 3. Under **A4**, if the stateful wrapper accepts the proof with $z_{i}$ equal to its current canonical state, it makes $z_{j}$ the unique canonical successor; a proof carrying the same stale $z_{i}$ cannot subsequently settle a competing endpoint.*

**Proof.**  Binding KZG openings and transcript-bound challenges fix the accumulator data and public boundaries supplied to the Decider. By Decider and folding soundness, an accepted proof implies a satisfying sequence of step instances for the relation in (1), except by violating **A2** or **A3**. Each satisfying step enforces the registered device opening and complete event tag, the physical guards, the deterministic update, and the history/sequence link. These equalities establish relation membership. Their key-holder authentication interpretation and the separate outsider-forgery bound are given in Theorem 3, subject to **A1**. Therefore, the accepted boundaries admit the witness sequence required by Definition 3. This establishes path soundness for either settlement or historical verification.

For a state-advancing invocation, Algorithm 2 additionally checks that $z_{i}$ equals the current canonical state and that both boundaries carry the registered $pk_{\mathrm{dev}}$. The contract replaces $z_{i}$ with $z_{j}$ in the same atomic execution that accepts the proof. A later proof carrying the stale $z_{i}$ fails the canonical-state comparison, so two competing successors cannot be accepted sequentially unless **A4** fails. Thus, any successful non-reachable or competing settlement contradicts at least one stated assumption.    □

Table 5 summarizes the security properties, enforcement mechanisms, and validation evidence.

### 5.2. Event Authenticity and Strict Signal Binding

The data-decoupling threat in Section 3.2 arises when a prover authenticates or validates one event but commits another. HeRZ uses the same allocated variables $\left(q_{t+1},\Delta_{t},V_{t},\eta_{t},\tau_{t},r_{t}\right)$ in the authentication tag and history update, and the relevant subset in the physical guards and deterministic state update. These circuit wires cannot be reassigned between checks. Consequently, authentication, policy validation, and state evolution refer to one event/order tuple in every satisfying step.

#### 5.2.1. Chosen-Message Security Notion

For k←KeyGen, give the adversary the public anchor $a=H_{P}\left(k\right)$ and adaptive oracle access to ${\mathrm{Tag}}_{k}$. The adversary wins EUF-CMA if it outputs $\left(m^{*},\sigma^{*}\right)$ with ${\mathrm{Verify}}_{k}\left(m^{*},\sigma^{*}\right)=1$ and $m^{*}$ never submitted to the tag oracle. Here *m* includes the salt, and the anchor and role are fixed; replay of a queried context is handled separately by the sequence and canonical-state checks. The auxiliary-anchor PRF assumption bounds by $\epsilon_{\mathrm{PRF}}$ the advantage in distinguishing ${\mathrm{Tag}}_{k}$ from a random function $F:F_{p}^{6}\to F_{p}$, with the same public anchor and public hash access in both experiments. This is an explicit assumption on the concrete keyed construction, not a consequence of unkeyed collision resistance. For one final forgery attempt,

(33) $${\mathrm{Adv}}_{\mathrm{Tag}}^{\text{EUF-CMA}}\left(A\right)\leq \epsilon_{\mathrm{PRF}}+\frac{1}{p}.$$

Replacing the tag oracle by F costs at most $\epsilon_{\mathrm{PRF}}$; at an unqueried context, its field output is uniform, so a guessed tag succeeds with probability 1/p.

#### 5.2.2. Ideal-Hash Justification of the Fixed-Format Interface

To make the assumption’s rationale explicit, replace $H_{P}$ by a random oracle H on the distinct supported input schemas, following the ideal-model methodology of [41]. Sample *k* uniformly from $F_{p}\setminus \left\{0\right\}$ and publish a=H(k). A classical adversary may request arbitrarily chosen tags and make at most $Q_{H}$ public hash queries. In a hybrid experiment, sample the anchor and each new context’s tag independently, and couple these values to H at *k* and $\left(d_{\mathrm{auth}},k,a,m\right)$, respectively. The views agree unless a public query guesses *k* in the anchor or authentication schema. Each such query nominates at most one key; in the independent hybrid, its candidate is independent of the uniformly sampled secret. A union bound gives probability at most $Q_{H}/\left(p-1\right)$ for this event. At a fresh context, the remaining tag is uniform, giving

(34) $${\mathrm{Adv}}_{\mathrm{ideal}}^{\text{EUF-CMA}}\left(A\right)\leq \frac{Q_{H}}{p-1}+\frac{1}{p}.$$

This argument accounts for the public anchor, secret placement, and fixed role encodings. Its random-oracle idealization is not a proof that the concrete Poseidon sponge is a PRF: **A1** retains that instantiation assumption, informed by the Poseidon and keyed-sponge analyses [32,35]. The 1/p term is an ideal tag-guessing probability, not a concrete security-bit claim; constraint-rejection tests validate implementation behavior rather than this assumption.

**Theorem** **3** (Input Integrity Anchor)**.** *Every satisfying step uses one secret opening of the registered anchor to reproduce the authentication tag on the same event/order tuple used by its guards, update, and history. Under **A1**, an adversary without that secret, with the chosen-message access defined above, produces a valid tag for a previously unqueried context with probability at most $\epsilon_{\mathrm{PRF}}+1/p$. Source authenticity is interpreted within the acquisition domain of Section 3.1.*

**Proof.**  Constraint (8) and the shared secret wire bind the tag computation to an opening of the registered anchor. Constraint (10) and the shared event wires enforce the first statement. The anchor’s preimage and second-preimage resistance exclude recovering or substituting that opening under the stated model. The outsider-forgery statement follows from Equation (33), completing the connection between device binding and the common transition input.    □

### 5.3. State Continuity and Policy Enforcement

Replay and forking attacks are handled at two levels. Within a batch, the step circuit advances the five-field state and enforces $q_{t+1}=q_{t}+1$ and $\tau_{t}={\bar{\tau }}_{t}+1$, while folding requires every step to consume its predecessor’s output. Across batches, the wrapper requires the proof’s $z_{0}$ to equal the currently stored state before atomically replacing it with $z_{N}$. Consequently, a previously settled proof or a competing branch cannot be accepted after the canonical state has advanced.

Historical verification binds both boundaries and $N_{ij}$ to one satisfying sequence without advancing state. Checkpoint anchoring excludes self-chosen endpoints, while Proposition 1 composes adjacent verified segments.

HeRZ does not claim to know the absolute physical truth of the environment. Instead, it narrows the admissible space of cryptographic truth by embedding domain guards into $C_{\mathrm{step}}$. The range, voltage, nighttime, and Boolean constraints reject events that violate the modeled PV operating envelope even if they are otherwise device-bound. These constraints therefore enforce policy compliance for authenticated inputs rather than replacing trusted sensing or calibration.

### 5.4. Raw-Event Non-Disclosure and Leakage Boundary

HeRZ avoids placing raw per-event values, salts, authentication tags, or intermediate witnesses directly on-chain, and the Groth16 component proves the Decider relation without revealing its private witness. The settlement nevertheless exposes the five-field public state and the verifier transcript required for finality. In particular, the cumulative value, event count, and latest logical slot are public. Moreover, the evaluated KZG instantiation is binding but non-hiding, so its commitments, evaluations, and opening proofs are not a formal concealment mechanism and may disclose algebraic information about committed vectors. HeRZ therefore claims raw-event non-publication rather than end-to-end transcript privacy; aggregate inference, formal leakage quantification, and hiding commitments remain outside its present guarantees.

## 6. Performance Evaluation

### 6.1. Implementation Setup

#### 6.1.1. Cryptographic Framework and Environment

The Rust implementation uses Sonobe 9414c45, Arkworks 0.5.0, and the pinned ark-r1cs-std fork 440ca3, with the complete Nova/CycleFold Decider enabled and matching setup parameters. Nova uses the BN254–Grumpkin cycle, with KZG and Groth16 finalization; curve and Poseidon parameters are specified in Section 4.1.2 and Section 4.2.3. The step circuit enforces the authenticated five-field transition defined in Section 4.1.

Measurements use a Linux 6.8.0 server with two Intel Xeon E5-2680 v4 processors and 256 GiB RAM. One proving job runs at a time, with 14 workers pinned to logical CPUs 0–13 and 28–41. Rust/Cargo 1.97.1 and LLVM 22.1.6 use the default optimized release profile. The PLONK reference uses Node.js 20.20.2 and snarkjs 0.7.5. Solidity 0.8.24 optimized contracts are evaluated in REVM 19.7.0, not a live consensus network. Supplementary Materials S11/S13 list the compiler options, resolved features, dependency corrections, and parameter-cache details.

#### 6.1.2. Dataset and Execution Pipeline

Functional tests use hourly photovoltaic generation records supplied by Oriensol Technology Company Limited from Shanxi, China, for the supplied study period February–December 2024. Only generation is measured. The authorized aggregate audit found 333 ordered, distinct date rows with 7992 nonempty hourly values and two missing calendar dates; a non-date row adds 24 empty cells. The loader flattens rows and substitutes zero for missing/unparsable cells without reconstructing missing dates, yielding 8016 positions, not 8016 measurements. The first 100 control positions contain no empty cells. Security tests convert kWh to integer Wh by rounding after multiplication by $10^{3}$; logical slots follow row/hour order. Station capacity, calibration, and measured voltage/context are unavailable.

Voltage and day/night context are synthetic test fields. The harness marks hours 20–23 and 0–5 as night, assigns 210,000 mV at night and 220,000+(τmod10,000) mV otherwise, and sets valid nighttime increments to zero. It constructs 100 controls and 100 cases in each of four invalid-input classes; these are controlled relation tests, not independently observed field anomalies. S13 in the Supplementary Materials distinguishes this preprocessing from the earlier demonstration loader.

Performance and historical-audit measurements instead use independent synthetic histories: for index *t*, night means tmod24∉[6,19], energy is zero at night and 10(tmod100) Wh otherwise, voltage follows the same night/day rule, and logical slots are t+1. The scalability fixture uses test-only key 7 and salts 17(t+1)+3. At each *N*, all methods receive identical physical fields, and HeRZ/FlatZK also share authenticated inputs and boundary states. Every run generates a fresh complete proof; historical-audit histories use separately sampled nonzero keys and salts. No confidential trace is used for these resource measurements.

#### 6.1.3. Baseline Configurations

**Stateful FlatZK** is the equivalent non-recursive baseline: one Groth16 circuit [40] invokes the exact HeRZ step relation *N* times and proves $\left(N,z_{0},z_{N}\right)$, including all five fields at both boundaries. Its wrapper applies the same canonical-predecessor, atomic-update, checkpoint, and audit rules. Its circuit-specific key depends on *N*.

**Stateful Batch-PLONK** [42] is an additional resource reference. Its proof-bound adapter reconstructs the full boundary tuple from nine public fields, the shared device anchor, and circuit-specific *N*, then enforces canonical settlement. Its arity-specific Poseidon hashes and comparator input-width assumptions differ from the Arkworks relation; it is not the equivalent baseline. S13 in the Supplementary Materials lists these differences and the binding regressions.

**Oracle-ECDSA** submits one signed event per transaction; **Batch-Merkle** commits a root and batch size without proving transition policies. Their retained ABI/gas records provide submission-granularity context, not equivalent ZK state semantics.

#### 6.1.4. Measurement Protocol

Complete online time covers input preparation, validated parameter loading, and fresh proof generation through serialization. Peak RSS is the process high-water mark over that interval, including resident parameters and workers. Setup is measured in separate processes and cached compatibly for every method; native/EVM verification is reported separately. HeRZ includes all folds and the full Decider, FlatZK the monolithic circuit/prover, and PLONK witness preparation plus interleaved key reads/proving. Nested synthesis, Groth16, and KZG stages are not added twice. The OS page cache is not forcibly cleared.

The formal matrix comprises three methods at N=100,500,1000,2000, with five independent complete online runs each (60 runs), without outlier exclusion. Supplementary Materials S1–S6 report the means, sample SDs, medians, approximate 95% Student-*t* intervals (four degrees of freedom), and individual records. Setup remains a single observation per configuration; archived pilots (S12) are excluded. Historical audits retain their distinct resident-parameter scope (Section 6.2.4).

### 6.2. Experimental Results

#### 6.2.1. On-Chain Verification Cost and Scalability

**Figure 4:**
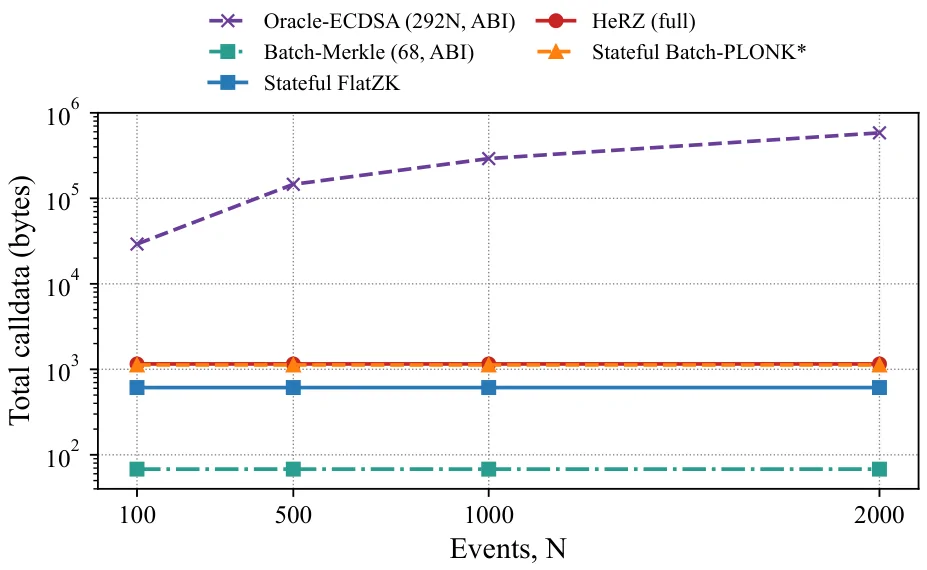
Total calldata for per-event submission and one batch settlement. Oracle/Merkle are ABI-derived context with different verified statements; ZK markers use the recorded full-configuration payloads. * Different-relation resource reference.

Figure 4 contrasts 292N bytes for *N* signed Oracle transactions with one 68-byte Merkle root commitment and a fixed-size ZK settlement. These payloads exclude transaction envelopes and network overhead. The source ABI and retained logs are documented in S17 in the Supplementary Materials. Root commitment alone does not prove policy-compliant evolution.

HeRZ’s compressed Decider proof is 384 bytes; complete canonical settlement uses 1156-byte calldata, compared with 612 bytes for equivalent FlatZK and 1124 bytes for the PLONK reference. First-settlement EVM execution costs are approximately 999.7, 416.8, and 438–439 kgas, respectively, excluding transaction-intrinsic gas. For HeRZ/FlatZK, underlying verification accounts for 870.9/288.1 kgas; the remaining 128.8 kgas covers canonical checks, writes, and logs. Historical audit uses the same proof and an additional version argument, giving 1188-byte calldata. Constant payload size is shared by these ZK configurations; HeRZ combines it with incremental construction.

#### 6.2.2. Proving Efficiency

**Figure 5:**
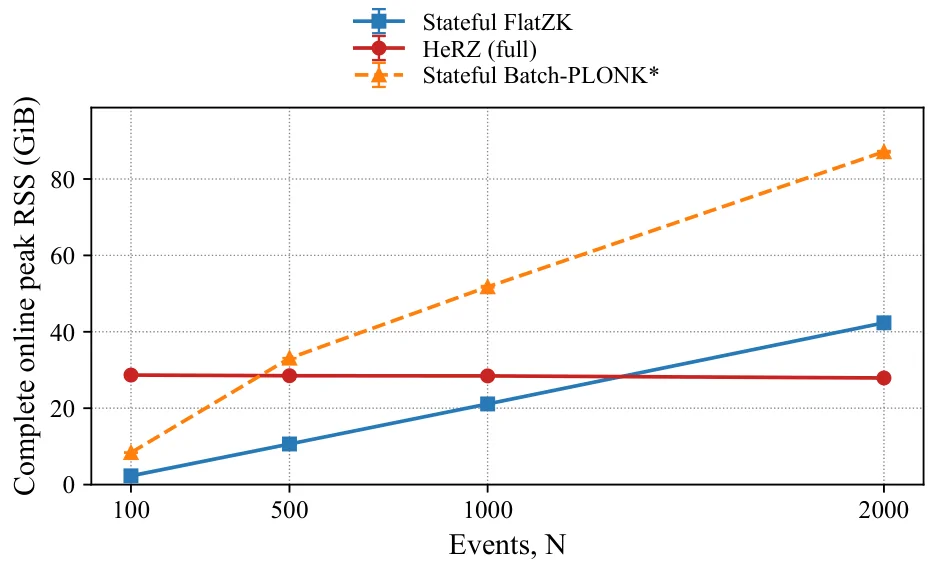
Complete online peak RSS: mean ± sample SD of five formal runs per configuration. Small SD bars may lie within markers. * Different-relation resource reference. Measurement scope is defined in Section 6.1.4.

**Figure 6:**
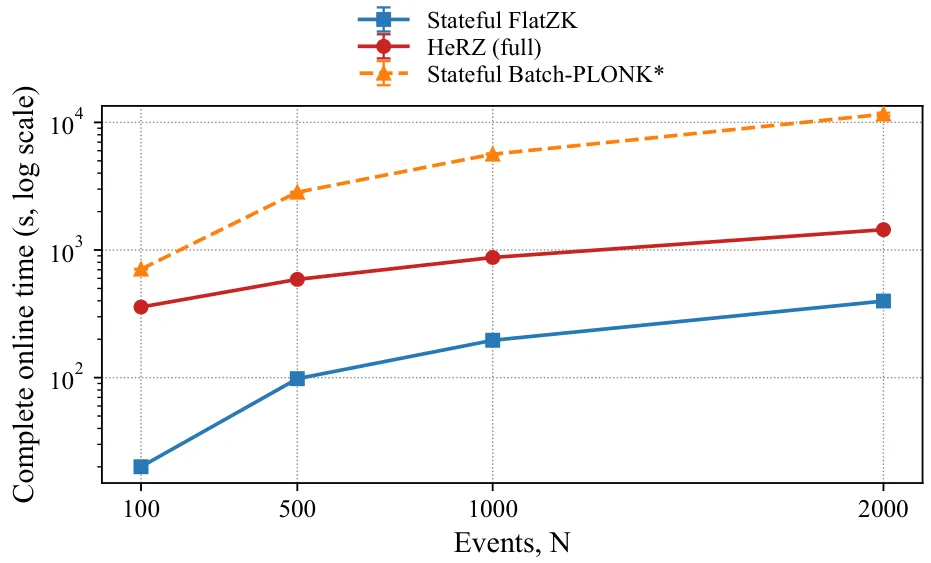
Complete online time on a logarithmic vertical axis, including input preparation and parameter loading. All points are five-run formal means with sample SD bars, following Figure 5. * Different-relation resource reference.

Table 6 and Figure 5 and Figure 6 show HeRZ’s mean complete online peak RSS at 27.92–28.68 GiB over N=100–2000, while equivalent FlatZK grows from 2.29 to 42.35 GiB. At N=2000, HeRZ uses 34.1% less peak memory at 3.63 times the online time. FlatZK is faster at every tested scale and uses less memory through N=1000. The memory ranking therefore reverses between the tested lengths 1000 and 2000, without establishing an exact crossover or strictly constant complete process memory.

HeRZ combines largely length-independent finalization with event-dependent folding (Table 7): at N=500, folding costs 0.571 s/event.

The separate setup observations are 140.97 s/19.74 GiB for HeRZ and, at N=2000, 282.86 s/34.50 GiB for FlatZK and 16165 s/58.46 GiB for PLONK preprocessing with an existing SRS. Supplementary Materials S4–S7 give all the setup, loading, and stage costs; historical PLONK setup at smaller scales is qualified because its thread/affinity settings are not established.

Against the PLONK resource reference (Section 6.1.3), HeRZ has lower complete online time at all four scales and lower peak memory from N=500 onward. At N=2000, the reference requires 11603.03 s and 87.14 GiB, versus HeRZ’s 1444.50 s and 27.92 GiB.

#### 6.2.3. Circuit Complexity Analysis

**Figure 7:**
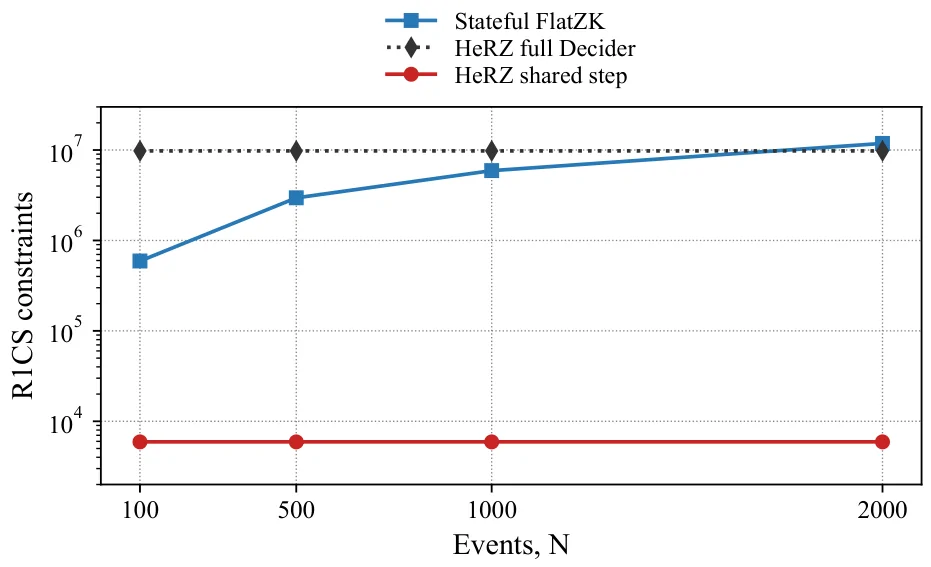
Shared-step, full Decider, and monolithic FlatZK R1CS counts on a logarithmic scale.

The shared step has 5924 R1CS constraints. Stateful FlatZK has 5924N+6, while the complete HeRZ Decider has 9,782,952 at every evaluated length (Figure 7). This fixed finalization circuit explains the gap between incremental-state and full-prover resources. Separately, PLONK key headers report 568,009/2,840,009/5,680,009/11,360,009 Plonkish constraints at N=100/500/1000/2000, with domain powers 20/22/23/24. These are not R1CS counts.

#### 6.2.4. Historical-Audit Cost and Parameter Reuse

Algorithm 3 is evaluated on an 80-event synthetic history, first anchored by actual settlements at checkpoint spacings 10 or 20. Queries start at non-genesis sequence $q_{i}=20$ and cover 20, 40, or 60 events, including cross-batch intervals. Five independently randomized proofs per configuration give 30 successful Rust/EVM verifications, all preserving the canonical state.

Table 8 combines the query and prerequisite costs. The audit proof time is 147.88–172.94 s with 34.45–34.51 GiB process RSS; it includes initialization, folding, and complete Decider proving with resident parameters but excludes initial loading. This retained measurement scope is not pooled with the fresh-process comparison. Reconstruction adds 14.08–42.47 ms; verification costs 885.226–885.290 kgas. Finer checkpoints increase selectable boundary density and preparation cost, while both settings retain the full witness history. Digest payload is not an Ethereum node’s physical storage footprint. Supplementary Materials S8–S10 provide the variability, reconstruction, and preparation details.

The same fixed-version proof configuration also supports different interval lengths: one deployed Decider accepts proofs for N=10,100,500,1000,2000, using identical folding parameters and Decider PK/VK. After four actual settlements have anchored sequences 20/40/60/80, the deployed Decider accepts audits [20,40] and [20,80] without state changes; incorrect *N*, boundaries, or versions are rejected. These results demonstrate reuse of a single fixed-version finalizer for variable-length settlement and anchored historical audits. FlatZK’s evaluated exact-length keys vary with *N*; PLONK supports universal SRS reuse within capacity but has circuit-specific preprocessing/VKs here. S7 in the Supplementary Materials provides the artifact digests and distinguishes these reuse layers.

#### 6.2.5. Security Validation Against Representative BCPS Attacks

The production step accepts all 100 trace-derived controls and rejects 400 constructed violations, grouped in Table 9. An authentication/time-only input control accepts range/night-policy violations while still rejecting forgery and tampering; it omits state updates and is not an end-to-end protocol ablation.

The synthetic suite adds 33 boundary, mutation, and encoding configurations with 32 trials each (1056 checks), cross-checking native behavior against R1CS. All match their expected outcomes, including acceptance of salt reuse with fresh context and authenticated in-envelope fabrication. Near-modulus cases retain modular arithmetic; the no-wrap interpretation in Section 3.3 and the wrapper’s canonical encodings/counter ordering remain distinct requirements.

Three complete Decider proofs for [0,4], [4,8], and [0,8] support 37 EVM cases covering two settlements, competing histories, public/proof mutations, and anchored audits. Cryptographic mutations are submitted directly to the verifier; separate wrapper tests check canonical state, endpoints, and versions. Valid forward settlements alone update state; repeated and post-revocation audits remain read-only. Missing witnesses and invalid endpoints are rejected. Table 9 summarizes the layers; S14 in the Supplementary Materials presents the detailed failure locations, counts, and mapping to Table 5. These tests validate implementation behavior rather than prove the cryptographic assumptions.

### 6.3. Performance Analysis

HeRZ decouples event collection from the final verifier transaction: off-chain work processes *N* transitions, while one settlement consumes fixed-width boundaries and a succinct proof. Figure 4 illustrates the communication consequence; the following theorem formalizes the on-chain bound. The full gas/payload comparison, including the earlier Oracle, Merkle, and isolated-batch context, is provided in S17 in the Supplementary Materials.

**Theorem** **4** (HeRZ Constant-Cost Physical Finality)**.** *The on-chain gas cost of verifying a HeRZ Physical State Commitment is O(1) with respect to the number of folded physical events N. Specifically,*

(35) $${Gas}_{HeRZ}=G_{tx/abi}+G_{acc}+G_{KZG}+G_{G16}+G_{state}=O\left(1\right)$$

*where $G_{tx/abi}$ denotes the transaction, calldata, opaque ABI unpacking, and self-call dispatch overhead; $G_{acc}$ denotes the accumulator reconstruction and public-input assembly; $G_{KZG}$ denotes the two KZG opening checks; and G_G16_ denotes the Groth16 Decider verification. The additional $G_{state}$ covers the fixed-width predecessor and version checks, canonical-state writes, checkpoint registration, and logs. Each term has a bound determined by the verifier and wrapper rather than by N; exact gas can vary with calldata byte values and storage-access state.*

**Proof.**  The compressed proof and accumulator openings have fixed size regardless of *N*. The IVC state also has fixed width |z|=5, carrying cumulative value, history hash, registered anchor, event sequence, and latest logical slot. The generated verifier’s KZG openings, Decider inputs, and wrapper arguments are fixed by the circuit and ABI rather than by the number of folded events. Hence every on-chain component has size independent of *N*, and the settlement gas is O(1) with respect to *N*.    □

**Corollary** **1** (Constant-Cost Historical Interval Verification)**.** *For anchored fixed-width checkpoints $z_{i}$ and $z_{j}$, the cryptographic cost and proof size for verifying $\Pi_{\left[i,j\right]}$ are O(1) with respect to $N_{ij}$. The implemented audit interface adds two fixed-width checkpoint-digest lookups and version/boundary checks, without an on-chain loop over the interval’s events or a state write.*

This bound concerns dependence on event count, not identical gas for every byte pattern. The equivalent FlatZK baseline also has fixed-size settlement; the architectural distinction lies in incremental proof construction, as quantified in Section 6.

## 7. Discussion

### 7.1. Trust and Deployment Boundaries

VSEI establishes an authenticated, policy-constrained trajectory under the acquisition and cryptographic assumptions of Section 3.1 and Section 5. A compromised key-holding acquisition domain can fabricate admissible readings; guards constrain their policy envelope, not physical truth. Calibration, trustworthy acquisition/time sources, and tamper resistance therefore remain deployment responsibilities. Raw events are not published, but public aggregates and non-hiding KZG transcripts retain the leakage boundary already specified in Section 5.

The measured complete prover requires server-class finalization resources, not a microcontroller or demonstrated high-rate real-time service. Incremental processing with scheduled finalization is the evaluated operating model. Safety-critical deployment additionally needs archive availability, key recovery, production setup governance, and live-network finality/failure handling; untrusted relays can still withhold messages.

### 7.2. Key and Policy Lifecycle

The owner registers an anchor and may terminally revoke it; Algorithm 2 rejects later settlements under a revoked anchor. Rotation/recovery creates a new key epoch and separately authorized asset association, not automatic continuity with the old trajectory. Distinct keys are required for each asset/chain/contract/epoch because the tag does not contain chain or contract identifiers. S15 in the Supplementary Materials details these controls and procedures.

A protocol version fixes the step relation, policy constants, cryptographic parameters, and immutable verifier. Changing the circuit or VK requires a new deployment; privately chosen thresholds cannot change the compiled policy. Old checkpoints and witnesses remain bound to their original version, allowing historical audit after prospective revocation but not cross-version recursion. Migration and setup trust require separate authorization and validation.

Policy extension is possible without changing this lifecycle: a separately tested irradiance–energy guard illustrates a richer cross-signal predicate. Its seven synthetic checks and illustrative coefficient are reported in S16 in the Supplementary Materials and are not included in the main circuit or claimed as a calibrated PV model.

### 7.3. Auditability and Resource Trade-Offs

HeRZ supports reporting-window and lifecycle audits by reconstructing the selected retained interval under Algorithm 3. Finer checkpoints improve endpoint choice at additional anchoring cost; they cannot recover discarded witnesses. The fixed-width accumulator, full finalizer, linear witness archive, and checkpoint registry are separate resource obligations. Constant-size verification does not imply constant reconstruction time or total storage.

The equivalent FlatZK comparison isolates proving architecture rather than stronger state validity: HeRZ incrementally preserves the same relation and reuses its finalizer, paying additional time and fixed costs for slower measured peak-memory growth. The evaluation compares one complete interval proof per method; segmented or padded non-recursive constructions are outside this comparison. Parameter reuse supports HeRZ’s existing incremental and audit paths, while the main contribution remains canonical, device-bound state evolution.

## 8. Conclusions and Future Work

HeRZ makes an authenticated asset-state trajectory, rather than an isolated report, the BCPS verification object. Its shared relation binds device identity, policy predicates, ordered history, and deterministic updates; canonical settlement consumes the current predecessor, while read-only audit verifies retained intervals between same-version checkpoints. The full implementation produces 384-byte compressed proofs and 1156-byte settlement calldata. Across 100–2000 events, mean complete online peak memory remains 27.92–28.68 GiB. At 2000 events, HeRZ saves 34.1% peak memory against the equivalent stateful FlatZK baseline at 3.63 times its online time, establishing the measured long-interval time–memory trade-off. Anchored audits and layered tests validate the implemented state semantics; parameter/verifier reuse supports incremental construction and selected historical queries. These results establish an executable path from sensor events to canonically settled and retrospectively auditable asset histories. Future work will study lower-cost complete finalization, multi-device evolution, adaptive policies, overlapping-interval proof reuse, and privacy-preserving commitments.

## Figures and Tables

**Table 1:** Representative related works and their verification targets.

Work	Verified Object or Mechanism	Relation to HeRZ
Nova/CycleFold [21,31]	Incremental computation through folding and curve-cycle checks	Supplies recursive machinery; the BCPS relation and ledger policy remain application choices.
Burgos and Pustišek [18]	IoT signature batches; Nova/Risc0 and Layer-2 authentication records	Closest recursive IoT authentication setting; the public batch statement differs from canonical asset-state reachability.
zk-IoT [17]	Chained firmware computations and device interactions using functional commitments	Verifies intermediate computation states; HeRZ specifies canonical asset-state consumption and historical boundary semantics.
TP-MCS [19]	Private truth-discovery aggregation using ZK arguments and Merkle commitments	Verifies sensing computation; HeRZ tracks an ordered, device-bound state trajectory.

**Table 2:** Protocol-level comparison of recursive IoT authentication and asset-state evolution.

Dimension	IoT Signature Aggregation [18]	HeRZ	Stateful FlatZK
Verified statement	Signature-batch authentication and membership	Device-bound, policy-compliant boundary-to-boundary evolution	Same step relation and public boundaries as HeRZ
Recursion	Nova; Risc0 also evaluated	Nova/CycleFold	None; one monolithic Groth16 circuit
Cross-batch continuity	Batch identifiers and ledger records; asset transition consumption not specified	Atomic current-predecessor consumption	Same atomic predecessor check
Interval audit	Batch membership; asset-boundary reconstruction not specified	Any retained, same-version anchored interval of at least two events	Same boundary policy; regenerate the interval circuit/proof
Witness retention	Aggregator receives signed data; interval archive policy not specified	Ordered private witnesses and checkpoint states	Same retained interval witnesses
Setup	Transparent proving configurations reported	KZG SRS and circuit-specific Groth16 setup	Circuit-specific Groth16 setup for each batch length
Data availability	Off-chain aggregation with ledger authentication records	Protected archive; proof alone does not recover events	Protected archive; proof alone does not recover events
Privacy	ZK batch authentication	Raw events not published; boundary aggregates and KZG transcript public	Same public application state
Policy updates	BCPS policy-epoch continuity not specified	New deployment/version; no cross-version recursive audit	Same version-specific wrapper policy

“Not specified” refers to the compared asset-state functionality, not an impossibility result. The non-recursive column is a controlled implementation baseline, not a claim that these application properties require folding.

**Table 3:** Key notation.

Variable	Description
$sk_{\mathrm{dev}},pk_{\mathrm{dev}}$	Symmetric device secret and registered hash anchor, $pk_{\mathrm{dev}}=H_{P}\left(sk_{\mathrm{dev}}\right)$
$E_{t}$	Private physical event $\left(\Delta_{t},V_{t},\eta_{t},\tau_{t}\right)$ at step *t*
$\Delta_{t},V_{t},\eta_{t}$	Energy increment, voltage, and nighttime indicator
$\tau_{t},r_{t},\sigma_{t}$	Logical acquisition slot, salt, and one-field-element symmetric Poseidon tag
$\Delta_{\max},V_{\max}$	Protocol-fixed upper bounds for energy increment and voltage
$H_{P},F_{p},d_{\mathrm{auth}},d_{\mathrm{hist}}$	Poseidon hash, BN254 scalar field, and hash-domain tags
$z_{t}$	IVC public state $\left({\mathrm{acc}}_{t},H_{t},pk_{\mathrm{dev}},q_{t},{\bar{\tau }}_{t}\right)$
$q_{t},{\bar{\tau }}_{t}$	Settled event sequence and latest logical time slot
${\mathrm{acc}}_{t},H_{t}$	Cumulative value and history hash after *t* events
$\omega_{t},F_{t},I_{t},R_{\mathrm{HeRZ}}$	Auxiliary event witness, transition map, temporal invariant, and HeRZ relation
$C_{\mathrm{step}},C_{\mathrm{dec}}$	Atomic step circuit and final Decider circuit
$N,\left(u_{t},w_{t}\right),\left(U_{t},W_{t}\right)$	Event count, incoming R1CS pair, and running relaxed R1CS pair
$\left[i,j\right],N_{ij},\Pi_{\left[i,j\right]}$	Selected evolution interval, its length, and its boundary-to-boundary proof
v,Digest(v,z)	Deployment version and checkpoint digest binding that version to the full state
$\bar{W},\bar{E},\bar{T},\rho_{t}$	KZG commitments, folding cross-term commitment, and transcript challenge
$\chi_{•},v_{•},\pi_{•}^{\mathrm{KZG}}$	KZG challenge, evaluation, and proof for •∈{W,E}
$H\left(vp\right),\mathrm{PSC},\Pi_{\mathrm{HeRZ}},\pi_{\mathrm{HeRZ}}$	Parameter digest, state commitment, settlement proof, and Decider SNARK

**Table 4:** Parameters and encodings of the evaluated authentication construction.

Item	Specification
Field	BN254 scalar field $F_{p}$, $\lceil \log_{2}p\rceil =254$; the scalar prime is given below.
Permutation	Width 5; rate 4 and capacity 1 field element; S-box $x^{5}$; 8 full rounds (four at each end) and 60 partial rounds.
Round constants/MDS	Arkworks 0.5.0 find_poseidon_ark_and_mds::<Fr>(254, 4, 8, 60, 0): Grain-LFSR generation, 68×5 round constants and a 5×5 MDS matrix; no matrix skipped.
Hash interface	Arkworks CRH::<Fr>::evaluate: initialize a fresh zero state, absorb the ordered field-element slice, then squeeze one native field element. No byte-string concatenation, length field, or application-level padding is added.
Fixed schemas	Anchor: 1 field; authentication: 9 fields in Equation (5); history: 10 fields in Equation (13). Variable-length inputs are not accepted by these protocol interfaces.
Role constants	$d_{\mathrm{auth}}=0x4845525A41555448$ (HERZAUTH); $d_{\mathrm{hist}}=0x4845525A48495354$ (HERZHIST), each converted directly from its unsigned 64-bit integer to $F_{p}$.
Key, salt, and tag	Provisioning requirement: uniform nonzero secret; event salt: fresh uniform field element. Deterministic test fixtures exercise the circuit, not key provisioning. Tag: one untruncated field element (254-bit container, 32-byte canonical field serialization).
Encoding	Fixed-position canonical field elements; scaled measurements and logical counters use their nonnegative integer representatives below *p*. The salt is part of the authenticated context, not a substitute for a secret key.

p=21888242871839275222246405745257275088548364400416034343698204186575808495617.

**Table 5:** Security properties, enforcement mechanisms, and validation evidence.

Property	Protocol Enforcement	Analytical/Empirical Evidence
Device-bound event integrity	Registered anchor and symmetric full-event tag within the trusted acquisition domain	Theorem 3; device-binding constraint tests
Signal integrity	Shared circuit wires bind authentication, guards, update, and history	Theorem 3; post-authentication tampering tests
Policy compliance	Protocol-fixed range, voltage, context, and Boolean constraints	Policy-compliance tests and authentication/time-only input controls (Section 6.2.5).
Temporal continuity	Sequence/time advancement, history linkage, and relation-preserving folding	Theorem 2; skipped-slot integration tests
Replay/fork resistance	Canonical-state equality and atomic state replacement	Theorem 2; two-batch, stale, replay, and competing-valid-interval EVM tests
Path soundness	Full Decider binds the folded relation and both public boundaries	Theorem 2; KZG/Groth16 and public-input mutation tests
Interval auditability	Boundary-parametric proof with anchored endpoint checks	Algorithm 3; full-proof endpoint/version tests and read-only audits
Raw-event non-publication	Private step witnesses and zero-knowledge Decider proof	Public/private input analysis; leakage limitations stated below

**Table 6:** Complete online comparisons: five formal runs per configuration.

Scheme	*N*	Runs	Mean Online (s)	Mean Peak RSS (GiB)
HeRZ (full)	100	5	357.73	28.680
HeRZ (full)	500	5	589.11	28.509
HeRZ (full)	1000	5	875.10	28.459
HeRZ (full)	2000	5	1444.50	27.920
Stateful FlatZK	100	5	20.03	2.291
Stateful FlatZK	500	5	98.19	10.629
Stateful FlatZK	1000	5	196.27	21.110
Stateful FlatZK	2000	5	398.34	42.352
Stateful Batch-PLONK *	100	5	707.88	8.370
Stateful Batch-PLONK *	500	5	2839.51	33.096
Stateful Batch-PLONK *	1000	5	5637.94	51.773
Stateful Batch-PLONK *	2000	5	11603.03	87.135

* Different-relation resource reference. Sample SDs, medians, 95% confidence intervals, and individual records: Supplementary Materials S1–S3. Scope: Section 6.1.4.

**Table 7:** Main online stages at N=500 (mean seconds; five runs each).

Stage	HeRZ	FlatZK	PLONK *
Input preparation	0.515	0.513	0.673
Folding-parameter load and relation reconstruction	1.918	–	–
Validated proving-key load	165.158	29.996	–
Initialization	1.113	–	–
Folding	285.531	–	–
Complete Decider	134.868	–	–
Monolithic Groth16 proving	–	67.674	–
Witness preparation	–	–	3.321
Interleaved key/witness reads and proving	–	–	2835.515

* Resource reference; no separate load-only phase. Nested synthesis/Groth16/KZG and verification are detailed in S4 and S6 in the Supplementary Materials and are not added twice. Minor bookkeeping/serialization is included in complete online time, not separately itemized here.

**Table 8:** Historical queries and prerequisite checkpoint preparation.

Spacing	Length	Proof (s)	Peak RSS (GiB)	Audit (kgas)
10	20	147.88	34.472	885.281
10	40	159.83	34.452	885.283
10	60	172.94	34.514	885.250
20	20	148.06	34.506	885.226
20	40	159.93	34.480	885.276
20	60	171.78	34.459	885.290
**Spacing**	**Checkpoints/txs**	**Archive/Digests (B)**	**Prep. (s)**	**Prep. (kgas)**
10	9/8	33,656/288	1138.28	7518.95
20	5/4	33,624/160	592.40	3794.74

Query means use five proofs each, start at $q_{i}=20$, and preserve canonical state. Parameters are resident; loading is excluded. Preparation is once per 80-event history; checkpoint counts include genesis. Full scope and variability: Supplementary Materials S8–S10.

**Table 9:** Layered validation and the input-policy control.

Layer/Input	Cases	Observed Behavior
Trace-derived controls	100	All accepted under the compiled policy.
Constructed violations	400	Range, night generation, forgery, and tampering: 100 rejections each.
Synthetic relation/encoding	1056	All expected outcomes match, including valid boundaries, salt reuse, and in-envelope inputs.
Full-proof EVM	37	6 accepts/31 rejects; only two forward settlements update canonical state.
Auth./time-only control	–	Accepts range/night violations; rejects forgery/tampering. No state-update computation.

The case groups, failure locations, and mappings to Table 5 are provided in S14 in the Supplementary Materials. The control isolates input checks, not the complete protocol.

## Data Availability

The hourly photovoltaic generation records were provided by Oriensol Technology Company Limited and are not publicly available due to confidentiality restrictions. The experimental methodology and evaluation results are reported in this article and the accompanying numerical Supplementary Materials; the latter reports the independent synthetic workloads, not the confidential generation series.

## Supplementary Materials

The following supporting information can be downloaded at https://www.mdpi.com/article/10.3390/s26196226/s1. The accompanying files are as follows: HeRZ_Supplementary_Material.pdf (S1–S17: statistics, implementation, and validation); HeRZ_Supplementary_Data.zip (individual results, metadata, validation records, and independent synthetic workloads); and HeRZ_Reproducibility_Materials.zip (implementation and test code, dependency and configuration records, and statistical and plotting scripts). The numerical records distinguish the 60 formal online runs from the separately measured historical audits and archived pilots.
