# Supplementary material for: HeRZ: Hierarchical Recursive Zero-Knowledge Verification for Canonical and Auditable Asset-State Evolution in Blockchain-Cyber-Physical Systems
Source: Sensors (Basel). 2026 Sep 30;26(19):6226. doi: 10.3390/s26196226 (PMC13667571; doi:10.3390/s26196226)
Supplement: Supplementary file 1 [file sensors-26-06226-s001.zip › HeRZ_Supplementary_Material.pdf]

HeRZ Numerical Evaluation Supplement

The current scalability comparison uses the complete Decider and fresh-process online measurements. Historical-audit evidence is retained with its separate resident-process timing scope. All inputs here are synthetic; no confidential photovoltaic measurements, real device secrets or retained witness archives are included.

HeRZ-full and Stateful FlatZK enforce the same concrete event and canonical boundary relation. *Stateful PLONK denotes Stateful-Batch-PLONK, a resource reference with canonical settlement but a different Poseidon instantiation and integer-comparator assumptions. It is not a strictly equivalent event-relation baseline.

The complete formal matrix comprises three methods and four lengths (N=100, 500, 1000 and 2000), with five fresh-process complete-online runs per configuration, totaling 60. All formal attempts passed native/EVM verification and all five observations are retained; historical pilots are archived separately in S12.

SD is the sample standard deviation. The 95% Student-t interval estimates the arithmetic mean within this host/configuration (t = 2.776445 for five runs). Small-sample intervals are approximate and are not cross-deployment guarantees. A single observation has neither an estimated SD nor a confidence interval. The OS page cache is not forcibly cleared.

S1 Complete online time in seconds

Scheme	N	n	Mean	SD	Median	95% CI of mean
HeRZ-full	100	5	357.728	0.826	357.850	[356.703, 358.753]
HeRZ-full	500	5	589.108	3.582	589.445	[584.661, 593.556]
HeRZ-full	1000	5	875.100	1.869	875.376	[872.780, 877.420]
HeRZ-full	2000	5	1444.500	3.588	1443.407	[1440.046, 1448.955]
Stateful FlatZK	100	5	20.026	0.117	19.967	[19.880, 20.172]
Stateful FlatZK	500	5	98.189	0.159	98.199	[97.992, 98.386]
Stateful FlatZK	1000	5	196.272	1.000	196.439	[195.031, 197.513]
Stateful FlatZK	2000	5	398.339	7.820	396.364	[388.629, 408.049]
Stateful PLONK*	100	5	707.882	4.545	706.192	[702.238, 713.525]
Stateful PLONK*	500	5	2839.513	28.561	2846.838	[2804.050, 2874.975]
Stateful PLONK*	1000	5	5637.938	68.082	5632.686	[5553.404, 5722.473]
Stateful PLONK*	2000	5	11603.031	317.810	11465.982	[11208.418, 11997.645]

S2 Complete online peak RSS in GiB

Scheme	N	n	Mean	SD	Median	95% CI of mean
HeRZ-full	100	5	28.680	0.061	28.689	[28.605, 28.756]
HeRZ-full	500	5	28.509	0.168	28.557	[28.300, 28.718]
HeRZ-full	1000	5	28.459	0.320	28.623	[28.063, 28.856]
HeRZ-full	2000	5	27.920	0.163	27.907	[27.718, 28.122]
Stateful FlatZK	100	5	2.291	0.001	2.291	[2.290, 2.292]
Stateful FlatZK	500	5	10.629	0.042	10.615	[10.577, 10.681]
Stateful FlatZK	1000	5	21.110	0.044	21.099	[21.056, 21.165]
Stateful FlatZK	2000	5	42.352	0.040	42.363	[42.302, 42.402]
Stateful PLONK*	100	5	8.370	0.053	8.394	[8.304, 8.436]
Stateful PLONK*	500	5	33.096	0.035	33.089	[33.053, 33.139]
Stateful PLONK*	1000	5	51.773	0.208	51.659	[51.515, 52.030]
Stateful PLONK*	2000	5	87.135	0.204	87.237	[86.881, 87.389]

Complete online starts before input preparation and includes validated parameter loading, fresh witness/folding work and complete proof generation through serialization. Setup, native verification and EVM validation are outside that boundary. Peak memory is the measured process high-water RSS within the online interval, including necessary resident parameters and all worker threads.

S3 Individual formal measurements

Scheme	N	Run	Online s	Peak GiB	Native verify s	Settlement gas
Stateful FlatZK	1000	1	197.159273	21.061428	0.003357	416836
Stateful FlatZK	1000	2	197.041942	21.154163	0.003309	416836
Stateful FlatZK	1000	3	196.439252	21.098576	0.003357	416824
Stateful FlatZK	1000	4	196.039688	21.158653	0.003332	416848
Stateful FlatZK	1000	5	194.679436	21.078945	0.003366	416812
Stateful FlatZK	100	1	20.224384	2.290272	0.003348	416800
Stateful FlatZK	100	2	19.941475	2.292252	0.003343	416812
Stateful FlatZK	100	3	19.956096	2.291161	0.003337	416800
Stateful FlatZK	100	4	19.966786	2.291851	0.003347	416812
Stateful FlatZK	100	5	20.041243	2.291054	0.003333	416800
Stateful FlatZK	2000	1	411.949448	42.371017	0.003375	416860
Stateful FlatZK	2000	2	392.784039	42.300411	0.003373	416860
Stateful FlatZK	2000	3	397.049966	42.363117	0.003372	416836
Stateful FlatZK	2000	4	396.363940	42.323975	0.003326	416860
Stateful FlatZK	2000	5	393.547345	42.402161	0.003370	416824
Stateful FlatZK	500	1	98.193368	10.702366	0.003346	416860
Stateful FlatZK	500	2	97.947040	10.605713	0.003350	416848
Stateful FlatZK	500	3	98.211124	10.598988	0.003351	416860
Stateful FlatZK	500	4	98.393216	10.614578	0.003347	416836
Stateful FlatZK	500	5	98.198980	10.625248	0.003373	416860
HeRZ-full	1000	1	875.375896	28.724052	0.010178	999739
HeRZ-full	1000	2	873.962514	28.721737	0.010194	999727
HeRZ-full	1000	3	877.298659	28.070004	0.013280	999715
HeRZ-full	1000	4	876.284790	28.157612	0.013238	999703
HeRZ-full	1000	5	872.577869	28.623425	0.010626	999727
HeRZ-full	100	1	356.597512	28.579754	0.010574	999667
HeRZ-full	100	2	357.398345	28.715431	0.010626	999631
HeRZ-full	100	3	358.860667	28.681129	0.013256	999667
HeRZ-full	100	4	357.932303	28.688686	0.013270	999655
HeRZ-full	100	5	357.850405	28.737282	0.013268	999643
HeRZ-full	2000	1	1443.406897	27.893616	0.010637	999751
HeRZ-full	2000	2	1442.348040	27.680481	0.010765	999751
HeRZ-full	2000	3	1450.632996	27.907036	0.013247	999727
HeRZ-full	2000	4	1444.448706	28.126389	0.010591	999739
HeRZ-full	2000	5	1441.664304	27.991501	0.010710	999715
HeRZ-full	500	1	591.898895	28.556778	0.010091	999739

Scheme	N	Run	Online s	Peak GiB	Native verify s	Settlement gas
HeRZ-full	500	2	584.619039	28.250397	0.010193	999727
HeRZ-full	500	3	589.445380	28.588955	0.010119	999739
HeRZ-full	500	4	593.130752	28.454041	0.010109	999703
HeRZ-full	500	5	586.448035	28.695225	0.010178	999739
Stateful PLONK*	1000	1	5746.592264	52.047741	0.039747	438675
Stateful PLONK*	1000	2	5645.160985	51.938667	0.044373	439091
Stateful PLONK*	1000	3	5632.686146	51.571888	0.044349	438381
Stateful PLONK*	1000	4	5598.995791	51.646011	0.045820	437709
Stateful PLONK*	1000	5	5566.255116	51.658543	0.044027	438051
Stateful PLONK*	100	1	709.923184	8.403645	0.042314	437621
Stateful PLONK*	100	2	702.701501	8.301968	0.041696	439407
Stateful PLONK*	100	3	706.191671	8.425705	0.043699	437755
Stateful PLONK*	100	4	705.988753	8.326412	0.041708	437021
Stateful PLONK*	100	5	714.603622	8.393940	0.042843	436899
Stateful PLONK*	2000	1	12101.103571	87.305454	0.042712	438215
Stateful PLONK*	2000	2	11722.736644	87.236816	0.035804	438239
Stateful PLONK*	2000	3	11302.350269	86.813377	0.022200	438925
Stateful PLONK*	2000	4	11422.983791	87.052837	0.040976	439463
Stateful PLONK*	2000	5	11465.982430	87.266563	0.043162	437799
Stateful PLONK*	500	1	2821.421809	33.124596	0.042759	438585
Stateful PLONK*	500	2	2864.866532	33.060928	0.043588	439465
Stateful PLONK*	500	3	2799.563775	33.088501	0.020134	440139
Stateful PLONK*	500	4	2864.872895	33.066593	0.044172	438523
Stateful PLONK*	500	5	2846.838371	33.139366	0.019747	437091

The companion CSVs retain recorded precision, result-file provenance, workload and verifying-key digests. Each row is a new complete proof process, not repeated Decider evaluation of a cached final accumulator.

S4 Measured stages in seconds

Scheme	N	Stage	n	Mean s	SD s
HeRZ-full	100	complete online s	5	357.728	0.826
HeRZ-full	100	core prove s	5	192.183	1.125
HeRZ-full	100	decider s	5	134.426	1.212
HeRZ-full	100	fold s	5	56.681	0.132
HeRZ-full	100	folding parameter load and r1cs reconstruction s	5	1.965	0.021
HeRZ-full	100	groth16 inside decider s	5	133.753	1.216
HeRZ-full	100	init s	5	1.075	0.010
HeRZ-full	100	input s	5	0.131	0.005
HeRZ-full	100	kzg inside decider s	5	0.202	0.009
HeRZ-full	100	native verify s	5	0.012	0.001
HeRZ-full	100	parameter load s	5	163.447	0.670
HeRZ-full	100	step s inside init fold	5	1.479	0.007
HeRZ-full	100	synthesis inside groth16 s	5	29.832	0.198
HeRZ-full	500	complete online s	5	589.108	3.582
HeRZ-full	500	core prove s	5	421.512	1.888
HeRZ-full	500	decider s	5	134.868	1.151
HeRZ-full	500	fold s	5	285.531	0.943
HeRZ-full	500	folding parameter load and r1cs reconstruction s	5	1.918	0.012
HeRZ-full	500	groth16 inside decider s	5	134.211	1.192
HeRZ-full	500	init s	5	1.113	0.004
HeRZ-full	500	input s	5	0.515	0.002
HeRZ-full	500	kzg inside decider s	5	0.197	0.008
HeRZ-full	500	native verify s	5	0.010	0.000
HeRZ-full	500	parameter load s	5	165.158	2.457
HeRZ-full	500	step s inside init fold	5	7.405	0.024
HeRZ-full	500	synthesis inside groth16 s	5	29.709	0.147
HeRZ-full	1000	complete online s	5	875.100	1.869
HeRZ-full	1000	core prove s	5	707.953	1.345
HeRZ-full	1000	decider s	5	135.673	0.712
HeRZ-full	1000	fold s	5	571.186	1.108
HeRZ-full	1000	folding parameter load and r1cs reconstruction s	5	1.926	0.018
HeRZ-full	1000	groth16 inside decider s	5	134.917	0.711
HeRZ-full	1000	init s	5	1.094	0.006
HeRZ-full	1000	input s	5	0.991	0.008
HeRZ-full	1000	kzg inside decider s	5	0.202	0.007
HeRZ-full	1000	native verify s	5	0.012	0.002

Scheme	N	Stage	n	Mean s	SD s
HeRZ-full	1000	parameter load s	5	164.223	1.731
HeRZ-full	1000	step s inside init fold	5	14.818	0.049
HeRZ-full	1000	synthesis inside groth16 s	5	29.826	0.141
HeRZ-full	2000	complete online s	5	1444.500	3.588
HeRZ-full	2000	core prove s	5	1275.304	2.232
HeRZ-full	2000	decider s	5	134.572	1.596
HeRZ-full	2000	fold s	5	1139.677	2.387
HeRZ-full	2000	folding parameter load and r1cs reconstruction s	5	1.925	0.018
HeRZ-full	2000	groth16 inside decider s	5	133.840	1.542
HeRZ-full	2000	init s	5	1.055	0.019
HeRZ-full	2000	input s	5	1.964	0.010
HeRZ-full	2000	kzg inside decider s	5	0.208	0.016
HeRZ-full	2000	native verify s	5	0.011	0.001
HeRZ-full	2000	parameter load s	5	165.299	2.345
HeRZ-full	2000	step s inside init fold	5	29.589	0.253
HeRZ-full	2000	synthesis inside groth16 s	5	29.837	0.115
Stateful PLONK*	100	complete online s	5	707.882	4.545
Stateful PLONK*	100	input s	5	0.549	0.013
Stateful PLONK*	100	key read witness read plonk prove s	5	706.436	4.540
Stateful PLONK*	100	native verify s	5	0.042	0.001
Stateful PLONK*	100	vk export s	5	0.007	0.000
Stateful PLONK*	100	witness prepare s	5	0.895	0.020
Stateful PLONK*	500	complete online s	5	2839.513	28.561
Stateful PLONK*	500	input s	5	0.673	0.004
Stateful PLONK*	500	key read witness read plonk prove s	5	2835.515	28.573
Stateful PLONK*	500	native verify s	5	0.034	0.013
Stateful PLONK*	500	vk export s	5	0.006	0.002
Stateful PLONK*	500	witness prepare s	5	3.321	0.033
Stateful PLONK*	1000	complete online s	5	5637.938	68.082
Stateful PLONK*	1000	input s	5	0.823	0.008
Stateful PLONK*	1000	key read witness read plonk prove s	5	5630.611	68.030
Stateful PLONK*	1000	native verify s	5	0.044	0.002
Stateful PLONK*	1000	vk export s	5	0.007	0.001
Stateful PLONK*	1000	witness prepare s	5	6.500	0.494
Stateful PLONK*	2000	complete online s	5	11603.031	317.810
Stateful PLONK*	2000	input s	5	1.128	0.007
Stateful PLONK*	2000	key read witness read plonk prove s	5	11582.453	317.849

Scheme	N	Stage	n	Mean s	SD s
Stateful PLONK*	2000	native verify s	5	0.037	0.009
Stateful PLONK*	2000	vk export s	5	0.009	0.002
Stateful PLONK*	2000	witness prepare s	5	19.442	0.091
Stateful FlatZK	100	complete online s	5	20.026	0.117
Stateful FlatZK	100	core prove s	5	13.580	0.070
Stateful FlatZK	100	input s	5	0.127	0.008
Stateful FlatZK	100	native verify s	5	0.003	0.000
Stateful FlatZK	100	parameter load s	5	6.314	0.054
Stateful FlatZK	100	step s inside prove	5	1.662	0.011
Stateful FlatZK	100	synthesis inside prove s	5	1.663	0.011
Stateful FlatZK	500	complete online s	5	98.189	0.159
Stateful FlatZK	500	core prove s	5	67.674	0.526
Stateful FlatZK	500	input s	5	0.513	0.005
Stateful FlatZK	500	native verify s	5	0.003	0.000
Stateful FlatZK	500	parameter load s	5	29.996	0.423
Stateful FlatZK	500	step s inside prove	5	8.280	0.021
Stateful FlatZK	500	synthesis inside prove s	5	8.282	0.021
Stateful FlatZK	1000	complete online s	5	196.272	1.000
Stateful FlatZK	1000	core prove s	5	135.535	0.664
Stateful FlatZK	1000	input s	5	0.997	0.007
Stateful FlatZK	1000	native verify s	5	0.003	0.000
Stateful FlatZK	1000	parameter load s	5	59.733	0.753
Stateful FlatZK	1000	step s inside prove	5	16.739	0.064
Stateful FlatZK	1000	synthesis inside prove s	5	16.744	0.064
Stateful FlatZK	2000	complete online s	5	398.339	7.820
Stateful FlatZK	2000	core prove s	5	277.508	9.238
Stateful FlatZK	2000	input s	5	1.975	0.024
Stateful FlatZK	2000	native verify s	5	0.003	0.000
Stateful FlatZK	2000	parameter load s	5	118.846	2.785
Stateful FlatZK	2000	step s inside prove	5	33.604	0.113
Stateful FlatZK	2000	synthesis inside prove s	5	33.612	0.113

Groth16 and KZG are inside the complete Decider, and Decider synthesis is inside Groth16. Step synthesis is inside initialization/folding or monolithic proving. These nested rows must not be added twice. PLONK interleaves key/witness reading with proving; no artificial folding, Decider or load-only phase is assigned to it. The raw proving_start_elapsed_s field is a cumulative timestamp marker, not an additional phase duration. HeRZ transfers its owned key to the original Decider API, avoiding an unnecessary large-key clone without omitting checks.

S5 Setup and parameter representations

Scheme	N	Observations	Process s	Peak GiB	Scope
HeRZ-full	shared	1	140.970	19.745	separate setup process including serialization. same corrected relation and parameters, 14 workers
Stateful FlatZK	100	1	13.510	1.753	separate setup process including serialization. same corrected relation and parameters, 14 workers
Stateful FlatZK	500	1	65.590	8.629	separate setup process including serialization. same corrected relation and parameters, 14 workers
Stateful FlatZK	1000	1	130.140	17.253	separate setup process including serialization. same corrected relation and parameters, 14 workers
Stateful FlatZK	2000	1	282.860	34.502	separate input/setup/serialization/hash process. current 14-worker configuration
Stateful PLONK*	100	1	127.250	10.338	historical PLONK circuit preprocessing with existing SRS; not a new ceremony. historical setup; worker/affinity settings not established by this time log
Stateful PLONK*	500	1	1305.310	27.586	historical PLONK circuit preprocessing with existing SRS; not a new ceremony. historical setup; worker/affinity settings not established by this time log
Stateful PLONK*	1000	1	5432.000	33.672	separate circuit preprocessing with existing power-25 SRS; not a new ceremony. current 14-worker configuration; GNU time process scope
Stateful PLONK*	2000	1	16165.000	58.458	separate circuit preprocessing with existing power-25 SRS; not a new ceremony. current 14-worker configuration; GNU time process scope

Setup/preprocessing uses separate processes and is not charged to every online repetition. These are single setup observations, not five-run means. PLONK uses an existing universal SRS; circuit preprocessing does not imply a new trusted ceremony. PLONK N=100/500 setup logs are historical observations with unestablished worker/affinity settings; N=1000/2000 preprocessing used the current 14-worker configuration. GNU time reports the latter long durations to one-second resolution. FlatZK N=2000 process time includes input/serialization/hash; setup alone is 249.239 s.

Artifact	Compressed B	Uncompressed B	Checked conversion peak GiB
flat100.metadata	113460656	226921264	0.238
flat1000.metadata	1067477680	2134955312	2.203
flat500.metadata	533739952	1067479856	1.103
herz.metadata	2108288728	4216577400	4.348

A/B uncompressed parameter caches contain the same PK/VK values as the retained compressed originals. Loading still validates canonical encodings/points. Format-conversion cost is separate from setup and formal online repetitions; all conversion phase timings and digests are in setup_observations.csv and the parameter metadata.

S6 Verification payloads and per-event costs

Scheme	N	Verifier gas	Settlement gas	Calldata B	s/event	gas/event	B/event
HeRZ-full	100	870876.600	999652.600	1156.000	3.577	9996.526	11.560
HeRZ-full	500	870953.400	999729.400	1156.000	1.178	1999.459	2.312
HeRZ-full	1000	870946.200	999722.200	1156.000	0.875	999.722	1.156
HeRZ-full	2000	870960.600	999736.600	1156.000	0.722	499.868	0.578
Stateful FlatZK	100	288133.800	416804.800	612.000	0.200	4168.048	6.120
Stateful FlatZK	500	288181.800	416852.800	612.000	0.196	833.706	1.224
Stateful FlatZK	1000	288160.200	416831.200	612.000	0.196	416.831	0.612
Stateful FlatZK	2000	288177.000	416848.000	612.000	0.199	208.424	0.306
Stateful PLONK*	100	308949.600	437740.600	1124.000	7.079	4377.406	11.240
Stateful PLONK*	500	309969.600	438760.600	1124.000	5.679	877.521	2.248
Stateful PLONK*	1000	309590.400	438381.400	1124.000	5.638	438.381	1.124
Stateful PLONK*	2000	309737.200	438528.200	1124.000	5.802	219.264	0.562

Gas values are EVM execution costs, excluding transaction-intrinsic gas. Settlement includes the actual verifier call, predecessor checks, canonical-state update and checkpoint registration. The difference from verifier gas is total wrapper overhead, not an isolated state-write measurement. The CSV field peak_mib_per_event_reporting_ratio supplies peak RSS/N only as an accounting ratio, not incremental allocation or a smaller machine-memory requirement. Full peak RSS remains the primary resource measure. Compressed proof objects are 384 B for HeRZ and 128 B for FlatZK; their ABI proof portions are 800 B and 256 B. PLONK has a 768 B ABI proof; JSON file size is not a canonical compressed-proof size.

S7 Cross-length parameter and verifier reuse

Actual validation used one deployed full Decider, without setup, VK replacement or Decider redeployment. All listed proofs passed that same verifier. Each N and endpoint field mutation was rejected.

N	Verified	Changed public fields rejected	Verifier gas
10	True	11	870915
100	True	11	870891
500	True	11	870963
1000	True	11	870963
2000	True	11	870963

Audit start	Length	Anchored boundaries	Read only	Cross-version rejected
20	20	True	True	True
20	60	True	True	True

The same contract first accepted the recorded forward settlements before historical queries. The audit starts are non-genesis and the longer interval crosses settlement batches. Verifier and proof digests, full endpoints and anchoring records are in cross_length_reuse.json; parameter_reuse.csv records every run.

Method	SRS / preprocessing	PK and VK across N	Current deployed verifier
HeRZ-full	Fixed step, policy and complete Decider version; actual reuse evidence above	Same cached folding and Decider parameters; run digests recorded	One deployed Decider in the recorded cross-length test
Stateful FlatZK	Circuit-specific Groth16 setup for each exact N; caches reused per shape	Different N-specific keys	Generated code embeds each VK; this implementation uses a matching deployment
Stateful PLONK*	Universal SRS reusable within capacity; exact-N circuit preprocessing cached	N-specific zkey/VK, not a fresh universal ceremony per proof	Generated code embeds VK and adapter fixes N; matching deployment in this implementation

Generic verifiers supplied with externally selected keys, fixed-capacity padding, cached key families and multiple shorter proofs are alternative implementation choices. They are not measured here. The comparison does not claim monolithic systems are unable to advance long histories or that multiple-proof gas is necessarily higher.

Retained PLONK SRS	Capacity power	Ceremony power	File bytes
pot21_final.ptau	21	21	2415920565
powersOfTau28_hez_final_25.ptau	25	28	38654788760

The retained setup commands use the power-21 file for N=100 and the same power-25 file for N=500/1000/2000. Thus these particular cached keys do not demonstrate reuse of one identical SRS file across all four lengths. snarkjs checks that the circuit domain power does not exceed the SRS power and that the prepared section is present. The N=100/500/1000/2000 zkey headers report domain powers 20/22/23/24 and 568,009/2,840,009/5,680,009/11,360,009 Plonkish constraints, respectively; these are not R1CS counts. An adequately sized universal SRS can support multiple circuit lengths without a new ceremony. The metadata is capacity/provenance evidence, not an audit of historical SRS contributions. Post-run SHA256 digests of the new N=1000/2000 keys and reused SRS are in postrun_parameter_digests.json; their key sizes are 23,599,886,144 and 47,199,770,816 bytes. All formal parameter identities are in parameter_reuse.csv.

S8 Retained complete historical-audit measurements

These 30 full-configuration proofs are retained from the resident-parameter audit experiment, not pooled with S1-S4. Proof time includes initialization, folding, key clone and complete Decider, but excludes initial checked key loading and archive reconstruction. RSS includes parameters and allocator state retained after checkpoint preparation. Each selected interval is refolded with fresh proof randomness.

Spacing	Length	n	Proof mean s	SD s	Peak mean GiB	Audit mean gas
10	20	5	147.880	0.386	34.472	885280.8
10	40	5	159.828	1.312	34.452	885283.2
10	60	5	172.943	0.417	34.514	885249.6
20	20	5	148.058	0.887	34.506	885225.6
20	40	5	159.929	0.613	34.480	885276.0
20	60	5	171.785	0.984	34.459	885290.4

All intervals start at sequence 20 and end at 40, 60 or 80, after actual forward settlements anchor both boundaries. Every audit preserves canonical state and uses 1,188 B calldata. These are controlled synthetic histories, not independent field datasets.

S9 Individual historical-audit observations

Spacing	Length	Run	Proof s	Peak GiB	Audit gas
10	20	run1	147.401470	34.425521	885288
10	20	run2	148.077937	34.452652	885288
10	20	run3	148.394228	34.466533	885288
10	20	run4	147.630843	34.548656	885264
10	20	run5	147.894672	34.468884	885276
10	40	run1	160.055438	34.543133	885276
10	40	run2	160.328625	34.423809	885264
10	40	run3	161.324032	34.429596	885288
10	40	run4	159.679653	34.431595	885300
10	40	run5	157.749911	34.431377	885288
10	60	run1	172.790482	34.483066	885264
10	60	run2	173.153461	34.556759	885240
10	60	run3	172.277133	34.485279	885252
10	60	run4	173.227060	34.558491	885240
10	60	run5	173.266261	34.486096	885252
20	20	run1	148.121612	34.505707	885228
20	20	run2	149.081128	34.506012	885216
20	20	run3	146.798162	34.505478	885216
20	20	run4	147.647928	34.505715	885228
20	20	run5	148.641876	34.505718	885240
20	40	run1	160.238736	34.579650	885288
20	40	run2	159.783757	34.453339	885288
20	40	run3	159.905900	34.453766	885264
20	40	run4	160.687338	34.456657	885264
20	40	run5	159.029649	34.456021	885276
20	60	run1	170.481503	34.457336	885300
20	60	run2	171.236723	34.459175	885288
20	60	run3	171.864790	34.459911	885300
20	60	run4	172.284202	34.460754	885276
20	60	run5	173.056669	34.459564	885288

S10 Witness archives checkpoint preparation and reconstruction

Spacing	Checkpoints	Archive B	Digest B	Settlements	Prep. proof s	Prep. gas
10	9	33656	288	8	1138.279	7518952
20	5	33624	160	4	592.400	3794740

Each spacing uses one independently generated 80-event history. These forward preparation totals exclude setup/key loading and are not charged to each repeated audit. Digest bytes count 32-byte registry payloads, not the physical database footprint. Archives retain all intermediate states and private witnesses; storage grows with retained history and those archives are not included.

Spacing	Length	n	Reconstruction mean ms	SD ms	Median ms
10	20	5	14.0780	0.0728	14.0630
10	40	5	28.1344	0.1258	28.1020
10	60	5	42.4674	0.8924	42.1120
20	20	5	14.1882	0.0623	14.1810
20	40	5	28.1346	0.0658	28.1470
20	60	5	42.0640	0.2569	42.1920

S11 Coverage failed attempts and provenance

Scheme	N	Formal / 5	Pilots	Status
HeRZ-full	100	5	0	complete
HeRZ-full	500	5	0	complete
HeRZ-full	1000	5	1	complete
HeRZ-full	2000	5	1	complete
Stateful FlatZK	100	5	0	complete
Stateful FlatZK	500	5	0	complete
Stateful FlatZK	1000	5	1	complete
Stateful FlatZK	2000	5	1	complete
Stateful PLONK*	100	5	0	complete
Stateful PLONK*	500	5	0	complete
Stateful PLONK*	1000	5	0	complete
Stateful PLONK*	2000	5	0	complete

Attempt	Status	Exit	Reason
cache_folding_parameters	failed	1	Preparation path/file lookup failure; not a proof rejection
plonk_negative_encoding_probe	failed	1	Preparation path/file lookup failure; not a proof rejection
plonk_header100	failed	1	Preparation path/file lookup failure; not a proof rejection
plonk_header500	failed	1	Preparation path/file lookup failure; not a proof rejection
compile_plonk1000	failed	127	Preparation path/file lookup failure; not a proof rejection
compile_plonk2000	failed	127	Preparation path/file lookup failure; not a proof rejection

The full attempts CSV includes every dependency/budget skip and preparation failure as well as successful runs. Windows-to-Linux path errors occurred in preparation and their logs were retained; successful parameter artifacts were relocated without changing values. A server-alias clarification stopped the queue between tasks; no formal sample was discarded on performance grounds. A later local connection interruption stopped scheduling after a completed attempt; all 15 existing formal results were retained on resume. The final continuation completed all 34 outstanding formal runs and two separate preprocessing jobs; 60 formal runs are now valid with zero formal failures or exclusions. Older budget/dependency skips are history, not current missing configurations. A final duplicate archive filename was recovered without rerunning proofs. Resource-headroom safety stops, actual OOM, timeouts and unstarted budget skips are distinct statuses.

Configuration provenance

Records	Classification	Use
July 2026 sonobe formal runs 1-3 used for submitted resource figures	confirmed simplified configuration	retained historical evidence only; excluded from full-Decider curves
September corrected formal, resumed, historical-audit and exploratory results	confirmed full configuration	audit observations retained with their original scope; main resource values not pooled with fresh-process measurements
July 27 full_decider_smoke directory time/phase logs	configuration provenance not fully established for those historical executions	historical diagnostic context only, not formal measurements
Current scale_eval independent-process outputs	confirmed full configuration	formal outputs only enter corresponding formal statistics; resource pilots remain separate

The submitted reduced-check configuration omitted the CycleFold witness-commitment consistency and CycleFold relation checks. Those checks are restored in the full Decider, with regenerated matching parameters and verified proofs. The old approximately 1.64 GiB value is not reused or relabeled as a measured folding-only result. Older logs without sufficient build provenance remain unconfirmed.

S12 Archived resource pilots

Scheme	N	Online s	Peak GiB	R1CS constraints	Settlement gas
Stateful FlatZK	1000	195.109	21.167	5924006	416824
Stateful FlatZK	2000	409.956	42.384	11848006	416860
HeRZ-full	1000	875.255	28.595	9782952	999739
HeRZ-full	2000	1445.975	28.266	9782952	999739

These single-run pilots are retained only for provenance. They are not pooled with the 60 formal repetitions, not plotted in the current figures, and not used to support current performance conclusions. The completed five-run summaries in S1-S2 supersede their earlier exploratory comparison.

S13 Implementation and measurement details

These details accompany the condensed Implementation Setup in the manuscript. They retain configuration and preprocessing information; no new experiment or change to the verified relation was introduced during this presentation revision.

The submitted July benchmark explicitly enabled Sonobe light-test and reported 422,149 Decider R1CS constraints. In Sonobe 9414c45, that feature omits CycleFold witness allocation, Pedersen witness-commitment consistency, and non-native CycleFold relation satisfaction checks. Honest-prover local checks do not restore these guarantees to the final verifier. The corrected full Decider contains 9,782,952 R1CS constraints and uses regenerated matching parameters. Reduced-check measurements, including the earlier approximately 1.64 GiB result, are excluded rather than relabeled as folding-only measurements.

A local Pedersen-gadget correction uses standard scalar multiplication for each commitment component, including odd-length and zero-scalar cases. Forty-four boundary regressions and complete native/EVM proofs validate the repair. The proof system, curves, application state and trust boundary are unchanged. The retained July 27 diagnostic logs lack sufficient contemporaneous feature/binary evidence to classify their executed configuration conclusively; directory names or RSS alone are not treated as proof of full mode.

The controlled build uses Rust Edition 2021, rustc/Cargo 1.97.1, LLVM 22.1.6, Sonobe 9414c45, Arkworks 0.5.0 and the pinned ark-r1cs-std fork 440ca3. Cargo uses the optimized default release profile with no additional RUSTFLAGS. Solidity 0.8.24 uses --bin --optimize, without via-IR; REVM 19.7.0 uses its pinned LATEST specification. Node.js 20.20.2/snarkjs 0.7.5 use 14 field-arithmetic workers and a 64-GiB JavaScript old-space setting, which is not a total-RSS limit. Larger circuit shapes were compiled using Circom 2.2.3 with --r1cs --wasm. The host and common CPU affinity are specified in the manuscript and S11.

HeRZ transfers the owned proving key into the original Decider API, removing a redundant large-key clone without skipping validation. HeRZ and FlatZK both use checked uncompressed parameter caches: conversion is separate from setup and online loading, while online decoding still validates canonical encodings and points. HeRZ online includes relation reconstruction, initialization, every fold, validated finalizer-key loading and full Decider proving. Step synthesis is nested inside initialization/folding, Groth16 and KZG inside Decider, and Decider synthesis inside Groth16; these durations are not summed twice. PLONK interleaves key/witness reads and proving and is not assigned an artificial Decider or load-only phase. All required resident objects remain in measured RSS.

The historical-audit measurements in S8 use the earlier resident-parameter process: initialization, folding, a proving-key clone and full Decider are timed, but initial key loading and archive reconstruction are separate. Peak RSS includes the loaded finalizer and retained allocations after checkpoint preparation. This scope is retained explicitly and is not pooled with fresh-process scalability results. Nova preprocessing uses a reproducibility seed; Groth16 setup uses operating-system randomness. These local benchmark setup artifacts are not evidence of a production multiparty ceremony.

The functional security loader rounds kWh times 1,000 to Wh; the earlier demonstration loader truncated after scaling. Only rounding describes the reported trace-derived security cases. The first 100 control positions contain no empty values. Night-generation attacks use the first 100 night slots with constructed positive increments. Missing dates are not reconstructed, and zero substitution in the historical data loader is not a protocol-level authenticated gap-recovery mechanism. The independent scaling input_seed field is a workload label, not a consumed PRNG seed; events follow the deterministic formulas stated in the manuscript.

The Stateful Batch-PLONK adapter binds all eleven application public fields from nine circuit outputs plus circuit-specific N and the shared device anchor. It passes real-proof public-field mutation and replay checks. Its Circom arity-1/9/10 Poseidon functions differ from the Arkworks sponge, and its comparator lacks equivalent input-width constraints. A retagged negative field representative p-1 is accepted as an R1CS witness in the retained probe. No malicious on-chain proof was generated for that probe. Consequently this implementation is a resource reference, not the equivalent event-relation baseline; the difference is not a limitation of PLONK in principle.

S14 Layered correctness and adversarial validation

Table S14.1 preserves the trace-derived case breakdown. These are constructed controls/violations, not estimates of naturally occurring attack frequency.

Input class	Cases	Accept	Reject	Enforced predicate
Valid controls	100	100	0	Compiled valid-event policy
Range violation	100	0	100	Energy range guard
Night generation	100	0	100	Context guard
Device forgery	100	0	100	Registered-device binding
Event tampering	100	0	100	Full-event authentication binding

The authentication/time-only input control retains registered-device, full-event authentication and logical-time checks, but omits physical guards and state-update computation. It accepts range/night-policy violations and rejects device forgery/tampering. It isolates input predicates, not the full recursively proved state-transition protocol.

The additional synthetic suite has 33 configurations and 32 reproducible trials each (1,056 checks; seed 0x20260917). It covers zero, maximum, near-maximum and negative field representatives; all authenticated context fields; non-Boolean context; cross-device substitution; replay, rollback and skipped slots; and malformed encodings. Each record gives expected behavior, actual circuit/decoder behavior, first failure layer and the corresponding property in manuscript Table 5. Native behavior is cross-checked against R1CS constraints.

Accepted cases include salt reuse with a newly bound context and correctly authenticated in-envelope fabrication. Artificial near-modulus states preserve modular arithmetic; acceptance by the relation is recorded, not reclassified as rejection. The wrapper separately enforces canonical field encodings and increasing interval counters. Integer semantics retain the manuscript no-wrap condition. Finite tests do not prove the concrete MAC or SNARK assumptions.

Table S14.2 preserves the full-proof EVM groups. Three complete proofs cover [0,4], [4,8] and [0,8]. The first two establish two settlements; the third also supplies a competing valid genesis interval and a cross-batch audit.

Test group	Accept	Reject	Manuscript Table 5 property
Two settlements; stale, replay, out-of-order and competing interval	2	4	Replay/fork resistance
Non-genesis, cross-batch, repeated and post-revocation audits	4	0	Interval auditability
Encoding, counter, device, checkpoint and version boundaries	0	11	Interval auditability
KZG/Groth16, public state/count and commitment mutations	0	13	Path soundness
Truncated and empty proof calldata	0	2	Path soundness
Forward settlement after revocation	0	1	Device-bound event integrity

Mutations flip the low bit of one serialized field. Public-input, Groth16 and KZG mutations are submitted directly to the cryptographic verifier, preventing wrapper rejection from masking missing proof binding. Separate wrapper checks require the expected custom errors. Changed KZG evaluations/challenges fail KZG; changed Groth16 coordinates/public states fail Groth16. Malformed opening/commitment coordinates and truncated calldata revert without a diagnostic string; no unsupported failure cause is assigned.

Only valid forward settlements update canonical state; repeated/post-revocation audits and failures do not. Archive checks reject missing witnesses, unanchored endpoints, version mismatch and reconstructed endpoint mismatch. Nine library and 44 Pedersen regressions provide separate evidence. Detailed records are in the companion CSVs: layered_security_post_correction, evm_layered_security, evm_failure_details and proof_mutations.

S15 Key and policy lifecycle procedures

This section retains the full lifecycle detail summarized in Discussion. Implemented contract controls are distinguished from deployment procedures.

Operation	Control and effect
Registration	Implemented: the owner registers a previously unknown nonzero anchor and initializes its five-field state. Deployment procedure: verify asset ownership and provision the secret to the authorized sensor/prover domain. The prototype does not perform hardware attestation.
Revocation	Implemented: the owner permanently revokes an active anchor. Later settlements fail, including proofs generated before revocation. The anchor cannot be re-registered; previously finalized states remain recorded.
Rotation	Deployment procedure: revoke the old anchor, provision a fresh independent key and register its new anchor. This starts a new state trajectory; no atomic migration or cross-key-epoch continuity is proved. An old/new asset association requires separate authorization.
Compromise recovery	Deployment procedure: revoke the affected anchor, retain the last finalized checkpoint, replace/resecure sensing and proving, and start a new epoch. Revocation is prospective: it neither identifies pre-detection forgeries nor repairs their physical meaning.
Domain isolation	Provision a distinct independent key for each (asset, chain, verifier contract, key epoch). Tags lack chain/contract identifiers; role constants alone do not prevent cross-deployment replay. The registry does not enforce global key uniqueness.
Historical use	Finalized checkpoints remain evidence for their original epoch. Audit should report registration/revocation context, not reactivate a revoked identity or treat an old proof as new settlement. Cross-epoch continuity needs a separately authenticated migration record.

A protocol version fixes the step relation, policy constants, Poseidon/folding parameters, SRS and Decider VK. The verifier address is immutable; replacing the circuit or VK requires a separately deployed version. The namespace combines chain, wrapper and immutable verifier configuration, without adding a sixth application-state field. Private threshold witnesses remain constrained to compiled constants.

Old checkpoints and retained witnesses use their original version. Same-version audits may continue after prospective revocation because they authenticate accepted history. Cross-version requests are rejected. Migration needs authorized deployment association, fresh deployment-isolated keys and validation of the new setup; no recursive cross-version continuity is implemented. Deployment must establish setup trust and retain old verifier artifacts.

S16 Independent policy extension example

An illustrative one-hour guard requires irradiance G (W/m2) to satisfy 0 <= G <= 1500 and energy increment Delta (Wh) to satisfy 0 <= Delta <= 400G. The coefficient is an illustrative area-efficiency product, not fitted to the confidential station. A deployment must authenticate and commit G under a new policy version.

Energy (Wh)	Irradiance (W/m2)	Expected	Observed
0	0	Accept	Accept
1	0	Reject	Reject
399999	1000	Accept	Accept
400000	1000	Accept	Accept
400001	1000	Reject	Reject
600000	1500	Accept	Accept
0	1501	Reject	Reject

The seven R1CS checks were executed in the retained policy_extension test and all matched. They are independent of the measured production circuit, performance counts and PV dataset. The example demonstrates a possible cross-signal predicate, not a complete or calibrated PV physical model. The source and prior passing log were inspected during consolidation; no test was rerun.

S17 Communication sources and contextual costs

Figure 4 contrasts one signed transaction per event with one batch settlement. All sizes are calldata bytes, excluding transaction envelopes and network overhead. The Oracle submitReading ABI has six event fields plus v/r/s, each ABI word occupying 32 bytes, and a four-byte selector: 292 bytes/event, hence 292N total. Merkle commitBatch(bytes32,uint256) has two words plus selector: 68 bytes/batch. This root commitment does not prove event policies or state reachability.

The retained eval.rs ABI builders and July formal_run_1 CSV agree at N=10/50/100/200/500: Oracle totals 2,920/14,600/29,200/58,400/146,000 bytes and Merkle 68 bytes throughout. Figure 4 extends these unchanged ABI formulas to N=1000/2000 as calculated payloads, not new executed workloads. All three ZK curves use validated formal artifacts at N=100/500/1000/2000, five runs per configuration. Proving-time and memory values come from the complete formal matrix, not from ABI formulas.

Configuration	Gas	Calldata	Verified statement / scope
Batch-Merkle	66,680-66,692	68 B	Retained root-and-batch-size commitment only
Batch-PLONK, isolated	297.9-300.1 kgas	1,060 B	Retained isolated N-event relation, not canonical settlement
HeRZ full	about 999.7 kgas	1,156 B	Current full relation, canonical advance and checkpoint
Stateful FlatZK	about 416.8 kgas	612 B	Same application relation and canonical rules as HeRZ
Stateful Batch-PLONK reference	about 438-439 kgas	1,124 B	Current boundary adapter; different concrete event relation
Oracle-ECDSA	O(N)	292N B	Per-event signature and state-hash update

The retained Oracle gas values at N=10/50/100/200/500 are 416,560; 1,946,192; 3,858,364; 7,682,600; and 19,157,972, respectively. Historical context is not pooled with current full stateful timings or treated as an equivalent-security gas ranking. The current verifier/settlement split and actual sample counts remain in S6.

Figure-source provenance records hashes of the ABI source, retained context CSV and current summary/per-run CSVs. Figures 5/6 show five-run formal means with sample-SD bars; small bars may lie within markers. Figure 6 uses a logarithmic time axis; no pilot or reduced-check point is plotted. Figure 7 uses exact recorded R1CS counts on a log axis: step 5,924, full Decider 9,782,952 and FlatZK 5,924N+6. PLONK accounting units are separately reported, not plotted as R1CS. Current gas values in S6 are EVM execution costs, excluding transaction-intrinsic gas.
